# Association of hippocampal subfield volumes with prevalence, course and incidence of depressive symptoms: The Maastricht Study

**DOI:** 10.1192/bjp.2023.143

**Published:** 2024-02

**Authors:** Jennifer Monereo-Sánchez, Jacobus F. A. Jansen, Martin P. J. van Boxtel, Walter H. Backes, Sebastian Köhler, Coen D. A. Stehouwer, David E. J. Linden, Miranda T. Schram

**Affiliations:** School for Mental Health & Neuroscience, Faculty of Health, Medicine and Life Sciences, Maastricht University, the Netherlands; and Department of Radiology & Nuclear Medicine, Maastricht University Medical Center, the Netherlands; Alzheimer Centrum Limburg, Department of Psychiatry and Neuropsychology, School for Mental Health and Neuroscience, Faculty of Health, Medicine and Life Sciences, Maastricht University, the Netherlands; School for Mental Health & Neuroscience, Faculty of Health, Medicine and Life Sciences, Maastricht University, the Netherlands; Department of Radiology & Nuclear Medicine, Maastricht University Medical Center, the Netherlands; and School for Cardiovascular Diseases, Faculty of Health, Medicine and Life Sciences, Maastricht University, the Netherlands; School for Mental Health & Neuroscience, Faculty of Health, Medicine and Life Sciences, Maastricht University, the Netherlands; and Department of Psychiatry and Neuropsychology, Maastricht University Medical Center, the Netherlands; School for Cardiovascular Diseases, Faculty of Health, Medicine and Life Sciences, Maastricht University, the Netherlands; Department of Psychiatry and Neuropsychology, Maastricht University Medical Center, the Netherlands; and Department of Internal Medicine, Maastricht University Medical Center, the Netherlands; School for Mental Health & Neuroscience, Faculty of Health, Medicine and Life Sciences, Maastricht University, the Netherlands; School for Mental Health & Neuroscience, Faculty of Health, Medicine and Life Sciences, Maastricht University, the Netherlands; School for Cardiovascular Diseases, Faculty of Health, Medicine and Life Sciences, Maastricht University, the Netherlands; Department of Internal Medicine, Maastricht University Medical Center, the Netherlands; and Maastricht Heart + Vascular Center, Maastricht University Medical Center, the Netherlands

**Keywords:** Magnetic resonance imaging, depressive disorders, neuroanatomy, neuropathology, cognitive neuroscience

## Abstract

**Background:**

Late-life depression has been associated with volume changes of the hippocampus. However, little is known about its association with specific hippocampal subfields over time.

**Aims:**

We investigated whether hippocampal subfield volumes were associated with prevalence, course and incidence of depressive symptoms.

**Method:**

We extracted 12 hippocampal subfield volumes per hemisphere with FreeSurfer v6.0 using *T*_1_-weighted and fluid-attenuated inversion recovery 3T magnetic resonance images. Depressive symptoms were assessed at baseline and annually over 7 years of follow-up (9-item Patient Health Questionnaire). We used negative binominal, logistic, and Cox regression analyses, corrected for multiple comparisons, and adjusted for demographic, cardiovascular and lifestyle factors.

**Results:**

A total of *n* = 4174 participants were included (mean age 60.0 years, s.d. = 8.6, 51.8% female). Larger right hippocampal fissure volume was associated with prevalent depressive symptoms (odds ratio (OR) = 1.26, 95% CI 1.08–1.48). Larger bilateral hippocampal fissure (OR = 1.37–1.40, 95% CI 1.14–1.71), larger right molecular layer (OR = 1.51, 95% CI 1.14–2.00) and smaller right cornu ammonis (CA)3 volumes (OR = 0.61, 95% CI 0.48–0.79) were associated with prevalent depressive symptoms with a chronic course. No associations of hippocampal subfield volumes with incident depressive symptoms were found. Yet, lower left hippocampal amygdala transition area (HATA) volume was associated with incident depressive symptoms with chronic course (hazard ratio = 0.70, 95% CI 0.55–0.89).

**Conclusions:**

Differences in hippocampal fissure, molecular layer and CA volumes might co-occur or follow the onset of depressive symptoms, in particular with a chronic course. Smaller HATA was associated with an increased risk of incident (chronic) depression. Our results could capture a biological foundation for the development of chronic depressive symptoms, and stresses the need to discriminate subtypes of depression to unravel its biological underpinnings.

## Background

The contribution of structural brain changes to the prevalence, course and incidence of late-life depression is a key topic of psychiatric neuroscience. Neuroanatomical substrates of depression could play a major role in diagnosis, prognosis, stratification of depression subtypes and treatment monitoring. Although some robust associations have been identified previously, the field has not yet yielded information that is clinically applicable, and contributions to pathophysiological understanding have been limited. The most replicated finding among older adults has been an association between smaller hippocampus volume with depression.^1–4^ This association may be especially noticeable with a longer depression duration or a larger number of depressive episodes.^5–9^ Little is known about the temporality of this association. Yet one study suggested that longer duration and severity of depression lead to faster development of hippocampal atrophy.^10^ Conversely, there is insufficient longitudinal data available to assess whether hippocampal atrophy may precede incident depression.^11^

Further, given that the hippocampus is a heterogeneous structure, composed of several subfields, each of which is characterised by specific cellular composition and characteristic neurophysiology,^12^ one may expect that different hippocampal subfields might be differentially associated with depression pathophysiology. Whereas this has been explored previously,^5,6,13,14^ conflicting results have been presented, likely because of limited sample sizes and a lack of longitudinal data.

## Aim

The aim of the present study was to investigate the associations of hippocampal subfield volumes with prevalence, course and incidence of depressive symptoms using a large neuroimaging sample. Specifically, we investigated the associations of hippocampal subfield volumes and depressive symptoms at baseline, and the associations of hippocampal subfields volumes at baseline with depressive symptoms during follow-up. In both cases we further subdivided the analysis according to the course of depression, i.e. chronic or transient, and corrected the analysis for demographic, cardiovascular and lifestyle risk factors.

## Method

### Study population and design

We used data from The Maastricht Study, an observational prospective population-based cohort study. The rationale and methodology have been described previously.^15^ In brief, the study focuses on the aetiology, pathophysiology, complications and comorbidities of type 2 diabetes mellitus (T2DM), heart disease and other chronic conditions, and is characterised by an extensive phenotyping approach. All individuals aged between 40 and 75 years and living in the southern part of the Netherlands were eligible for participation. Participants were recruited through mass media campaigns, the municipal registries and the regional Diabetes Patient Registry via mailings. Recruitment was stratified according to known T2DM status, with an oversampling of individuals with T2DM, for reasons of efficiency. Baseline data were collected between November 2010 and January 2018. Lag time between magnetic resonance imaging (MRI) and depression assessment at baseline was 102 days (s.d. = 120).

The authors assert that all procedures contributing to this work comply with the ethical standards of the relevant national and institutional committees on human experimentation and with the Helsinki Declaration of 1975, as revised in 2008. All procedures involving human participants were approved by the institutional Medical Ethical Committee (NL31329.068.10) and the Minister of Health, Welfare and Sports of the Netherlands (Permit 131088-105234-PG). All participants gave written informed consent.

For the current analysis complete data was available from 4653 participants for cross-sectional analysis and 4154 participants for longitudinal analysis. Supplementary Figure 1 available at https://doi.org/10.1192/bjp.2023.143 shows the flow chart of the study population.

### Brain MRI

Brain images were acquired on a 3T clinical magnetic resonance scanner (MAGNETOM Prismafit, Siemens Healthineers GmbH) located at a dedicated scanning facility (Scannexus, Maastricht, the Netherlands) using a head/neck coil with 64 elements for parallel imaging. The MRI protocol included a three-dimensional *T*_1_-weighted (*T*_1_w) magnetisation-prepared rapid acquisition gradient echo (MPRAGE) sequence (repetition time/inversion time/echo time (TR/TI/TE) 2300/900/2.98 ms, 176 slices, 256 × 240 matrix size, 1.0 mm cubic reconstructed voxel size); and a fluid-attenuated inversion recovery (FLAIR) sequence (TR/TI/TE 5000/1800/394 ms, 176 slices, 512 × 512 matrix size, 0.49 × 0.49 × 1.0 mm reconstructed voxel size).

Brain segmentation was performed with FreeSurfer v6.0^16^ using both *T*_1_w and FLAIR images as input. The arguments ‘-FLAIRpial’ and ‘-3T’ were used to optimise segmentation quality. Brain segmentations with insufficient quality, i.e. Euler numbers below 1.5 quartile (−80 for left hemisphere and −68 for right hemisphere) were excluded.^17^ Hippocampal subfields^18^ were segmented using multispectral segmentation, yielding hippocampus total volume and 12 hippocampal subfields per hemisphere (Supplementary Table 1). All extracted volumes were *z*-transformed prior to statistical analysis with respect to the distribution in the complete sample (*n* = 4643). Results are depicted using hippocampal subfields maps, a legend for these maps can be found in Supplementary Figure 2.

### Depression

Depressive symptoms were assessed by a validated Dutch version of the 9-item Patient Health Questionnaire (PHQ-9)^19^ both at baseline and follow-up. Follow-up data was collected annually over a period of 7 years, i.e. each participant was asked to complete the PHQ-9 questionnaire once every year, up to 7 years. The PHQ-9^19^ is a self-administered questionnaire that assesses the presence of the nine symptoms for the DSM-IV criteria for a major depressive disorder on a four-point Likert scale ranging from 0 ‘not at all’ to 4 ‘nearly every day’.^20^ When one or two items were missing, the total score was calculated as 9 × (total points/9 − number of missing items) and rounded to the nearest integer. When more items were missing, the total score was scored as missing. A cut-off score of ≥10 is most often used as a dichotomous scoring system for defining clinically relevant depressive symptoms, with a good sensitivity (88%) and specificity (78%).^21^ The internal consistency of the PHQ-9 in The Maastricht Study was good (Cronbach's alpha = 0.82 without T2DM, and 0.87 with T2DM).^22^

There was a time lag between the baseline data collection and the date of the MRI scan. Therefore, the PHQ-9 score obtained closest to the date of the MRI scan, regardless of whether the assessment was before or after the scan, was chosen as the baseline score for each individual. Subsequent assessments were labelled as follow-up 1, follow-up 2, and so forth, based on the order in which they occurred after the baseline assessment.

Here, we use the term ‘prevalent depressive symptoms’ to indicate the use of PHQ-9 scores as a continuum at baseline. We use the term prevalent depression to indicate clinically relevant depressive symptoms (PHQ-9 ≥ 10) at baseline. We subdivided prevalent depression according to its course as:

- ‘prevalent depression with a chronic course’ i.e. clinically relevant depressive symptoms (PHQ-9 ≥ 10) at baseline and clinically relevant depressive symptoms (PHQ-9 ≥ 10) at at least one follow-up time; and
- ‘prevalent depression with a transient course’ i.e. clinically relevant depressive symptoms (PHQ-9 ≥ 10) at baseline and no clinically relevant depressive symptoms (PHQ-9 < 10) during follow-up.

We use the term ‘incident depression’ to indicate no clinically relevant depressive symptoms (PHQ-9 < 10) at baseline and presence of clinically relevant depressive symptoms (PHQ-9 ≥ 10) at at least one follow-up time. We subdivided incident depression according to its course as:

- ‘Incident depression with a chronic course’ i.e. no clinically relevant depressive symptoms (PHQ-9 < 10) at baseline and clinically relevant depressive symptoms (PHQ-9 ≥ 10) at two or more follow-up moments; or
- ‘incident depression with a transient course’, i.e. no clinically relevant depressive symptoms (PHQ-9 < 10) at baseline and clinically relevant depressive symptoms (PHQ-9 ≥ 10) at one follow-up.

We used the term, ‘no depression’ as the comparison group, and include those participants with no clinically relevant depressive symptoms (PHQ-9 < 10) at baseline and no clinically relevant depressive symptoms (PHQ-9 < 10) at follow-up.

### General characteristics and covariates

General characteristics and covariates were measured at baseline. Educational level (low, intermediate, high), history of cardiovascular disease (CVD), smoking status (never, current, former), alcohol consumption (none, low, high) were assessed by questionnaires.^15^ We measured, height, weight, waist circumference, office blood pressure, plasma lipid profile, and 24 h urinary albumin excretion (twice) as described elsewhere.^15^

To determine T2DM status, all participants (except those who used insulin) underwent a standardised seven-point oral glucose tolerance test after an overnight fast. Glucose metabolism status was defined according to the World Health Organization 2006 criteria.^23^ Participants were considered to have T2DM if they had a fasting blood glucose ≥7.0 mmol/L or a 2 h post load blood glucose ≥11.1 mmol/L or used oral glucose-lowering medication or insulin. Cholesterol lowering medication, glucose-lowering medication and use of antidepressants was assessed in a medication interview at baseline where generic name, dose and frequency were registered.

### Statistical analyses

General characteristics of the study population were evaluated using independent *t*-tests, or χ^2^-tests when appropriate.

A total of five research questions (RQs) were asked. Are hippocampal subfield volumes associated with:

- prevalent depressive symptoms (RQ1);
- clinically relevant depressive symptoms (RQ2);
- clinically relevant depressive symptoms according to its course (chronic and transient) (RQ3);
- incident clinically relevant depressive symptoms (RQ4); and
- incident clinically relevant depressive symptoms according to its course (chronic and transient) (RQ5)?

We used negative binomial regression on data from *n* = 4643 participants to answer RQ1; logistic regression on data from *n* = 4643 participants for RQ2; multimodal logistic regression on data from *n* = 4174 participants for RQ3; and Cox proportional hazards regression with time to event on the time axis on data from *n* = 4174 participants for RQ4 and RQ5. An overview of the research questions, participants and groups can be seen in Supplementary Figure 1.

Associations were adjusted for potential confounders in two different models: model 1, adjusted for total brain volume (when analysing total hippocampal volumes), or for total hippocampal volume (when analysing hippocampal subfields), MRI lag time, age and gender; and model 2, additionally adjusted for T2DM status, education level, waist circumference, history of CVD, total-to-high-density lipoprotein cholesterol ratio, use of alcohol and smoking status.

We studied the left and right hemispheres separately, and analysed 1 total hippocampal volume and 12 hippocampal subfields for each hemisphere. The hippocampal subfields are correlated with each other (Supplementary Table 2). For this reason, correction for multiple comparisons was done in accordance with the matrix spectral decomposition method.^24^ Based on the resulting eigenvalues, the obtained effective number was *n* = 13, therefore alpha was set at 0.05/13 = 0.0039.

Several sensitivity analyses were performed based on the fully adjusted model (model 2):

- we excluded individuals with type 2 diabetes to assess whether they drive the observed associations;
- we adjusted for antidepressant medication use;
- we excluded participants who used antidepressant medication;
- to restrict analyses to ‘*de novo*’ depression, we excluded participants who had a history of major depressive disorder diagnosis (assessed through the Mini-International Neuropsychiatric Interview^25^) before baseline;
- we additionally adjusted for cognitive status using Mini-Mental State Examination score.^26^

Finally, we tested whether these associations differed according to gender, and T2DM status, by use of interaction analyses.

All statistical analyses were performed in R 4.0.2 (2020-06-22); analytic code is available on request from the corresponding author.

## Results

### General characteristics of the study population

The cross-sectional study population (*n* = 4643) had a mean age of 60.0 years (s.d. = 8.6), and 51.8% were women, 229 participants had prevalent depression (PHQ-9 ≥ 10).

Table 1 shows the general characteristics of the study population for longitudinal analysis (*n* = 4174) stratified for depressive status. A total of 190 participants had prevalent depression, 141 of them had a chronic course during follow-up, and 49 had a transient course. Out of 3984 participants free of depression at baseline, 376 developed incident depression. Participants with no depression were more often men and had a better cardiovascular profile than those with prevalent or incident depression. Demographics of participants not included in this study because of missing data or bad segmentation quality can be seen in Supplementary Table 3.

**Table 1:** General characteristics of the study population (*n* = 4174) stratified for depressive status^a^

Characteristic	No prevalent depression *n* = 3608	Prevalent depression *n* = 190	*P*^b^	Incident depression *n* = 376	*P*^b^
Age, years: mean (s.d.) range	60.4 (8.4) 40.0–79.7	57.3 (8.2) 40.4–75.2	**<0.001**	59.1 (8.7) 41.3–78.1	**0.005**
Gender, % women	51.0	61.1	**0.007**	57.2	**0.022**
BMI, kg/m^2^: mean (s.d.) range	26.1 (3.9) 14.4–56.3	27.8 (5.0) 16.0–44.3	**<0.001**	27.3 (4.7) 18.1–47.9	**<0.001**
Waist, cm: mean (s.d.) range	92.4 (12.1) 59.5–148.0	95.7 (14.0) 62.0–133.8	**0.002**	95.3 (14.3) 64.9–142.0	**<0.001**
Educational level			**<0.001**		**0.017**
Low%	28.8	38.1		34.7	
Medium, %	28.4	33.3		29.5	
High, %	42.9	28.6		35.8	
Alcohol consumption			**<0.001**		**<0.001**
None, %	14.9	28.0		23.4	
Low, %	59.4	54.5		54.0	
High, %	25.7	17.5		22.6	
Smoking status			**<0.001**		**<0.001**
Never, %	40.7	40.2		37.5	
Former, %	49.3	40.7		46.1	
Current, %	10.1	19.0		16.4	
Partner, % yes	86.6	77.4	**<0.001**	78.7	**<0.001**
T2DM, % yes^c^	16.0	26.8	**<0.001**	25.3	**<0.001**
CVD, % yes	11.2	16.5	**0.026**	10.8	0.846
Hypertension (% yes)	46.9	52.1	0.157	51.3	0.098
Cholesterol ratio, mean (s.d.) range	3.5 (1.1) 1.0–11.7	3.7 (1.4) 1.9–9.6	0.066	3.7 (1.2) 1.3–9.2	0.049
Cholesterol medication, % yes	24.9	27.4	0.447	27.4	0.292
Antidepressants, % yes	4.6	27.9	**<0.001**	15.2	**<0.001**
History of depression, % yes	24.7	79.1	**<0.001**	51.8	**<0.001**
MMSE score, mean (s.d.) range	29.2 (1.1) 21.0–30.0	29.0 (1.1) 25.0–30.0	**0.036**	29.1 (1.1) 24.0–30.0	0.158

Bold indicates *P* < 0.05. BMI, body mass index; CVD, cardiovascular disease; MMSE, Mini-Mental State Examination; PHQ-9, Patient Health Questionnaire; T2DM, type 2 diabetes mellitus.

a. No depressive symptoms is no clinically relevant depressive symptoms at baseline nor at follow-up. Prevalent depression is clinically relevant depressive symptoms at baseline. Incident depression is no clinically relevant depressive symptoms at baseline and clinically relevant depressive symptoms at follow-up .

b. Compared with no clinically relevant depressive symptoms at baseline and follow-up.

c. The study is oversampled with individuals with type 2 diabetes by design.

### Hippocampal subfields and prevalent depression

We found no associations between total hippocampus volume and prevalent depressive symptoms (PHQ-9 score as a continuum; Supplementary Table 4). Larger volumes in bilateral molecular layer (left relative risk (RR) = 1.11, 95% CI 1.05–1.17 and right RR = 1.14, 95% CI 1.08–1.20) and right hippocampal fissure (RR = 1.06, 95% CI 1.02–1.11), and smaller volumes in right dentate gyrus (RR = 0.89, 95% CI 0.83–0.96) and right cornu ammonis (CA)2/3 (RR = 0.91, 95% CI 0.87–0.96) and CA4 (RR = 0.89, 95% CI 0.83–0.96) were significantly associated with depressive symptoms in model 1. The association between smaller volumes in CA4 and depressive symptoms remained significant (RR = 0.89, 95% CI 0.83–0.96) after full adjustment (model 2). Hippocampal subfields associations in model 2 are depicted in Supplementary Figure 3.

**Fig. 1:**
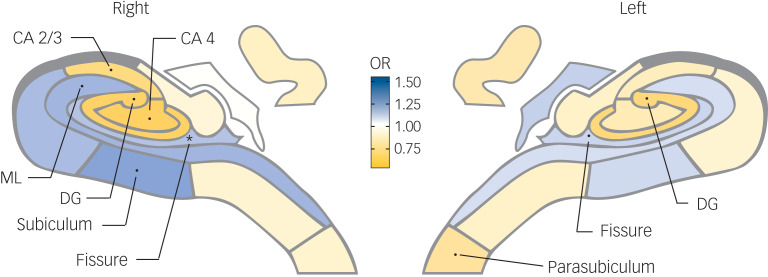
The hippocampal map shows the associations between hippocampal subfield volumes and prevalent depression (Patient Health Questionnaire (PHQ-9) ≥ 10). The diagram displays the subfields’ associations with prevalent depression, after full adjustment (model 2). The blue colour represents a positive association: a higher volume is associated with higher odds ratio (OR) for prevalent depression (PHQ-9 ≥ 10), whereas yellow represents a negative association. Dots show the hippocampal subfields with associations of *P* < 0.05; stars show the subfields that are significant after multiple comparison correction (*P* < 0.0039). See Supplementary Figure 2 for the hippocampal map legend. Prevalent depression is clinically relevant depressive symptoms (PHQ-9 ≥ 10) at baseline. CA, cornu ammonis; DG, dentate gyrus; ML, molecular layer.

No association of total hippocampus volume with prevalent depression (PHQ-9 ≥ 10) was found (Supplementary Table 5). A larger right molecular layer (odds ratio (OR) = 1.46, 95% CI 1.18–1.81) and hippocampal fissure (OR = 1.32, 95% CI 1.13–1.53) and smaller CA2/3 (OR = 0.72, 95% CI 0.59–0.87) were associated with prevalent depression in model 1. The association with right hippocampal fissure remained significant (OR = 1.26, 95% CI 1.08–1.48) after full adjustment (model 2). Results are depicted in Figure 1.

### Hippocampal subfields volumes and course of prevalent depression

**Fig. 2:**
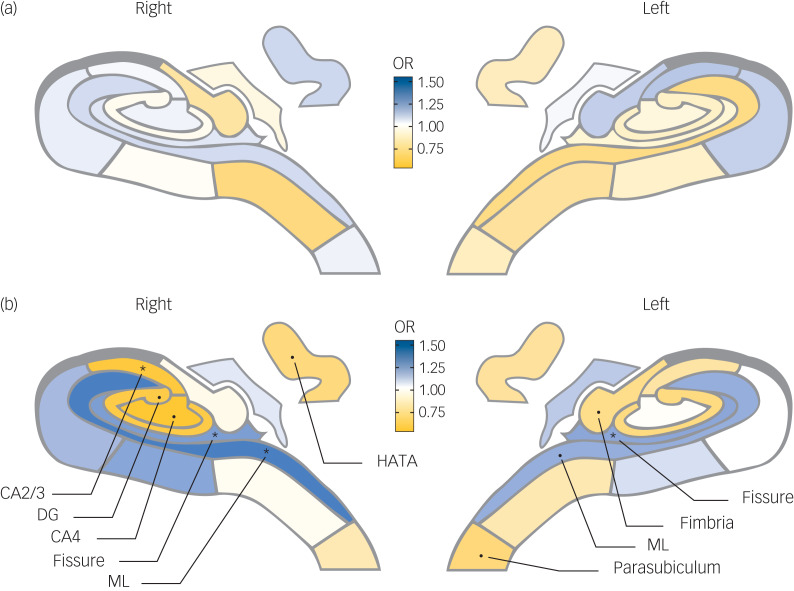
The hippocampal map shows the associations between hippocampal subfield volumes and (a) transient or (b) chronic course of prevalent depression. The diagrams display the subfields’ associations with (a) transient course of prevalent depression, and (b) chronic course of prevalent depression in model 2. The blue colour represents a positive association: a higher volume is associated with higher odds ratio (OR) for depression, whereas yellow represents a negative association. Dots show the subfields with associations of *P* < 0.05, stars show the subfields that are significant after multiple comparison correction (*P* < 0.0039). See Supplementary Figure 2 for the hippocampal map legend. Prevalent depression is clinically relevant depressive symptoms at baseline. CA, cornu ammonis; DG, dentate gyrus; HATA, hippocampal amygdala transition area; ML, molecular layer.

A significant association between lower volumes in the right hippocampus total volume and chronic course of prevalent depression (OR = 0.68, 95% CI 0.52–0.87) was found after full adjustment (model 2). Larger bilateral hippocampal fissure (left OR = 1.42, 95% CI 1.18–1.70 and right OR = 1.46, 95% CI 1.21–1.77) and molecular layer (left OR = 1.45, 95% CI 1.13–1.85 and right OR = 1.66, 95% CI 1.27–2.19), as well as smaller left parasubiculum (OR = 0.73, 95% CI 0.59–0.90) and right CA3 (OR = 0.60, 95% CI 0.47–0.77) were associated with a higher risk ratio of chronic course of prevalent depression in model 1. After full adjustment (model 2), higher volumes in bilateral hippocampal fissure (left OR = 1.37, 95% CI 1.14–1.64 and right OR = 1.40, 95% CI 1.15–1.71) and right molecular layer (OR = 1.51, 95% CI 1.14–2.00), as well as smaller volumes in right CA3 (OR = 0.61, 95% CI 0.48–0.79) remained significantly associated with the chronic course of prevalent depression. Results are depicted in Fig. 2, and details can be found in Supplementary Table 6. No significant associations were found for the transient course of prevalent depression.

### Hippocampal subfields and incident depression

No significant associations were found between hippocampal volumes and incident depression (Supplementary Table 7).

### Hippocampal subfields and course of incident depression

A statistically significant association between lower volume in left HATA and the chronic course of incident depression was found (hazard ratio (HR) = 0.70, 95% CI 0.55–0.89), whereas we found no associations with the transient course of incident depression (Supplementary Table 8).

### Sensitivity analysis

Sensitivity analysis show results with preserved direction of effect and higher *P*-values when (a) excluding participants with T2DM, (b) adjusting for antidepressant medication, (c) excluding participants using antidepressant medication, (d) excluding participants with a lifetime of major depressive disorder diagnosis and (e) adjusting for cognition.

Results are detailed for prevalent depressive symptoms (Supplementary Table 9), prevalent depression (Supplementary Table 10) and prevalent depression with a chronic course (Supplementary Table 11). No interactions with gender or T2DM were found in the associations of depression and hippocampal volumes (data not shown).

## Discussion

### Main findings

In this middle-to-older-aged population, we studied the associations between hippocampal subfield volumes and prevalence, course and incidence of depressive symptoms. We show that specific hippocampal subfields are associated with prevalent depression, especially with a chronic course. One subfield was also associated with incident depression, yet only when the course was chronic. To our knowledge, this is the first study that has investigated the association of specific hippocampal subfield volumes with depressive symptoms in a population-based sample.

### Comparison with findings from other studies

Larger right hippocampal fissure and bilateral molecular layer, as well as smaller right dentate gyrus, and CA3 and CA4, were associated with prevalent depressive symptoms (PHQ-9 score as a continuum). Larger right fissure and molecular layer, as well as smaller right CA3 were associated with prevalent depression (PHQ-9 ≥ 10), independently of age, gender and total hippocampal volume. The associations between hippocampal subfields and depression severity have been previously explored among patients with major depressive disorder (MDD) in small clinical samples (*n* = 41 to 163). In line with our results, Hu et al (2019)^27^ found that lower CA3 and CA4/dentate gyrus volumes were associated with more severe depressive symptoms. However, we could not replicate their findings of a significant association with lower volumes in the subiculum. In addition, our results are in line with previous studies in clinical samples that compared patients with MDD with controls,^6,14,28^ who found smaller volumes in CA structures, the subiculum and tail associated with MDD. A main difference is, however, that we found these associations more often in the right hemisphere, whereas the previous studies reported differences in both hemispheres.

These differences may be explained by the differences in study samples (clinical versus population based) and difference in instruments to assess depression (MDD diagnosis versus depressive symptoms). Although our definition of depression status is a reliable approach for MDD screening,^22,29^ our study sample likely includes less severely affected individuals. This might mean that CA1, the subiculum and tail have more subtle or later roles in depression pathophysiology, being only detectable in more severe depression, in line with results from Roddy et al.^6^

Further, we found an association with the hippocampal fissure volume, which has not been reported before. The hippocampal fissure is not a tissue structure *per se*, but a cerebrospinal fluid filled cavity, defined as a space between the dentate gyrus and the molecular layer.^18^ The possibility of a larger fissure volume being driven by the general atrophy of the hippocampus was considered. However, our findings, in agreement with Roddy et al,^6^ revealed an increase in volume specifically within the molecular layer, which does not align with this hypothesis. As an alternative explanation, we propose that the observed larger volume of the hippocampal fissure might be attributed to the reshaping of the hippocampus.

We further studied the association of hippocampal subfields with the course of prevalent depression. We found some hippocampal subfields were associated with a chronic course but none was associated with a transient course. Specifically, the larger bilateral fissure and molecular layer, and smaller volumes of left parasubiculum, right CA3 and right total hippocampal volume were associated with a chronic course. Previous studies found an association between depression recurrence and total hippocampal atrophy,^6,7,9^ yet only one study explored this association with hippocampal subfields, finding smaller volumes in dentate gyrus.^5^

The different patterns of hippocampal morphology in patients with transient or chronic depression may suggest that hippocampus atrophy is of importance in the pathophysiology of chronic depression but is not in transient depression. Some studies have also explored the utility of hippocampal subfields in the measurement of treatment response, with promising results finding an increase in hippocampal volumes after some treatments, and remission of depression.^27,30,31^ Overall, our results suggest that the different subfields of the hippocampus might have a different sensitivity to depression. Cytology studies suggest that a deficiency in neurotrophic support might be the cause,^32^ and that the compensation of neurotrophic factors through pharmacological therapy could reverse the pathological process of depression.^33^

We found no significant associations between hippocampal subfield volumes and risk of incident depression, in line with a previous smaller population-based study.^11^ Yet, when we subdivided this analysis according to the course, we found lower volumes in left HATA to be associated with incident depression with a chronic course. This might indicate that there is a different aetiology in incident depression with a chronic course versus a transient course. Replication of our findings is needed, and future studies should clarify whether changes in hippocampal volumes are specific for subtypes of depression.

### Strengths and limitations

Strengths of this study include the large sample size and population-based design, the extensive assessment of potential confounders that reduces the chance of residual confounding and the annual assessment of depressive symptoms over a 7-year period. To assess robustness of observed associations we performed a range of sensitivity analyses. Results remained similar after additionally adjusting for antidepressant medication, cognition and limiting the sample to *de novo* depression. Potential selection and/or attrition bias, which is inherent to prospective population-based studies, may have resulted in underestimation of the observed associations. In addition, depression was measured with the PHQ-9 questionnaire, which is a reliable and valid tool for the measurement of depressive symptoms, but is not equal to a clinical diagnosis of MDD.^19,34,35^ Additionally, the progression of depression was evaluated annually during follow-up sessions. This approach allows for the possibility that a person may have experienced depression at some point during the year but it might not have been present at the time of assessment. As a result, there is a chance of encountering false negatives in some participants.

Our study utilises a population-based cohort with an intentional oversampling of individuals with T2DM. In the general population, the lifetime prevalence of depression stands between 10% and 25% for women and from 5% to 12% for men,^36^ whereas within our sample it was 30% (1349 participants out of 4643 participants with cross-sectional data). This observed difference can be attributed to the deliberate oversampling of people with T2DM, as individuals with T2DM are almost twice as likely to experience depression.^36^ Consequently, the prevalence of depression is elevated within our sample. We corrected our analysis for T2DM, and excluded participants with T2DM in a sensitivity analysis. Yet, it is imperative to consider these aspects carefully when generalising the results of our study.

Finally, hippocampal volumes were extracted using the FreeSurfer v6.0 automated tool. FreeSurfer v6.0 has proven to be a reliable method for hippocampal subfields volume's measurement, showing a good agreement with manual segmentation.^37^ It also shows a good test–retest reliability, especially in the tail, subiculum, presubiculum, CA1–4, dentate gyrus and molecular layer.^38,39^ Moreover, its use has previously proved useful to provide insight into the neurobiological underpinnings of several brain-related traits and disorders.^40^ In this study, the hippocampal segmentation was implemented with the additional use of a FLAIR image (multispectral segmentation) which has shown to additionally improve subfields segmentation reliability.^18,41^ Further, all FreeSurfer output used in The Maastricht Study undergoes quality control through the exclusion of outliers based on Euler numbers, a technique that shows similar quality control benefits to visual inspection for hippocampal subfields segmentation,^17^ reinforcing the solidity of the data. However, it is crucial to interpret the results with caution, especially concerning the smaller subfields such as the hippocampal fissure. Despite utilising diverse techniques to improve the accuracy of hippocampal subfield segmentation, the inherent complexity and intricacies of these smaller subfields present challenges that demand careful consideration.

### Implications

In conclusion, differences in hippocampal volumes of specific subfields, indicating hippocampal atrophy, were associated with prevalent depression, in particular with a chronic course. In longitudinal analyses we found some evidence that smaller volume in the left HATA was associated with a risk of incident depression with a chronic course. Our results indicate that changes in hippocampus subfield volumes may co-occur or follow the onset of depressive symptoms, rather than precede it. We found limited evidence to support that specific volume changes could precede the onset of (chronic) depressive symptoms. Therefore, our results could be capturing a biological foundation for the development of chronic depression, and further stresses the need to discriminate between subtypes of depression to unravel its biological underpinnings.

## Supporting information

Monereo-Sánchez et al. supplementary material 1 Monereo-Sánchez et al. supplementary material

Monereo-Sánchez et al. supplementary material 2 Monereo-Sánchez et al. supplementary material

## Data Availability

The data used in this project is part of The Maastricht Study and is available on formal request from the corresponding author, M.T.S. Please visit https://www.demaastrichtstudie.nl/data-guidelines for more information.

## References

[1] Kempton MJ, Salvador Z, Munafò MR, Geddes JR, Simmons A, Frangou S, et al. Structural neuroimaging studies in major depressive disorder: meta-analysis and comparison with bipolar disorder. Arch Gen Psychiatry 2011; 68: 675–90. 21727252 10.1001/archgenpsychiatry.2011.60

[2] Zhang FF, Peng W, Sweeney JA, Jia ZY, Gong QY. Brain structure alterations in depression: psychoradiological evidence. CNS Neurosc Ther 2018; 24: 994–1003. 10.1111/cns.12835 PMC6489983 29508560

[3] Hickie I, Naismith S, Ward PB, Turner K, Scott E, Mitchell P, et al. Reduced hippocampal volumes and memory loss in patients with early-and late-onset depression. Br J Psychiatry 2005; 186: 197–202. 15738499 10.1192/bjp.186.3.197

[4] Sexton CE, Le Masurier M, Allan CL, Jenkinson M, McDermott L, Kalu UG, et al. Magnetic resonance imaging in late-life depression: vascular and glucocorticoid cascade hypotheses. Br J Psychiatry 2012; 201: 46–51. 22753853 10.1192/bjp.bp.111.105361

[5] Treadway MT, Waskom ML, Dillon DG, Holmes AJ, Park MTM, Chakravarty MM, et al. Illness progression, recent stress, and morphometry of hippocampal subfields and medial prefrontal cortex in major depression. Biol Psychiatry 2015; 77: 285–94. 25109665 10.1016/j.biopsych.2014.06.018 PMC4277904

[6] Roddy DW, Farrell C, Doolin K, Roman E, Tozzi L, Frodl T, et al. The hippocampus in depression: more than the sum of its parts? Advanced hippocampal substructure segmentation in depression. Biol Psychiatry 2019; 85: 487–97. 30528746 10.1016/j.biopsych.2018.08.021

[7] Campbell S, Marriott M, Nahmias C, MacQueen GM. Lower hippocampal volume in patients suffering from depression: a meta-analysis. Am J Psychiatry 2004; 161: 598–607. 15056502 10.1176/appi.ajp.161.4.598

[8] Geerlings MI, Gerritsen L. Late-life depression, hippocampal volumes, and hypothalamic-pituitary-adrenal axis regulation: a systematic review and meta-analysis. Biol Psychiatry 2017; 82: 339–50. 28318491 10.1016/j.biopsych.2016.12.032

[9] Kronmüller K-T, Pantel J, Köhler S, Victor D, Giesel F, Magnotta VA, et al. Hippocampal volume and 2-year outcome in depression. Br J Psychiatry 2008; 192: 472–3. 18515903 10.1192/bjp.bp.107.040378

[10] Taylor WD, McQuoid DR, Payne ME, Zannas AS, MacFall JR, Steffens DC. Hippocampus atrophy and the longitudinal course of late-life depression. Am J Geriatr Psychiatry 2014; 22: 1504–12. 24378256 10.1016/j.jagp.2013.11.004 PMC4031313

[11] den Heijer T, Tiemeier H, Luijendijk HJ, van der Lijn F, Koudstaal PJ, Hofman A, et al. A study of the bidirectional association between hippocampal volume on magnetic resonance imaging and depression in the elderly. Biol Psychiatry 2011; 70: 191–7. 21641582 10.1016/j.biopsych.2011.04.014

[12] Fanselow MS, Dong H-W. Are the dorsal and ventral hippocampus functionally distinct structures? Neuron 2010; 65: 7–19. 20152109 10.1016/j.neuron.2009.11.031 PMC2822727

[13] Lim HK, Hong SC, Jung WS, Ahn KJ, Won WY, Hahn C, et al. Automated hippocampal subfields segmentation in late life depression. J Affect Disord 2012; 143: 253–6. 22840623 10.1016/j.jad.2012.04.018

[14] Ballmaier M, Narr KL, Toga AW, Elderkin-Thompson V, Thompson PM, Hamilton L, et al. Hippocampal morphology and distinguishing late-onset from early-onset elderly depression. Am J Psychiatry 2008; 165: 229–37. 17986679 10.1176/appi.ajp.2007.07030506 PMC2834288

[15] Schram MT, Sep SJ, van der Kallen CJ, Dagnelie PC, Koster A, Schaper N, et al. The Maastricht study: an extensive phenotyping study on determinants of type 2 diabetes, its complications and its comorbidities. Eur J Epidemiol 2014; 29: 439–51. 24756374 10.1007/s10654-014-9889-0

[16] Fischl B. Freesurfer. Neuroimage 2012; 62: 774–81. 22248573 10.1016/j.neuroimage.2012.01.021 PMC3685476

[17] Monereo-Sánchez J, de Jong JJ, Drenthen GS, Beran M, Backes WH, Stehouwer CD, et al. Quality control strategies for brain MRI segmentation and parcellation: practical approaches and recommendations-insights from the Maastricht study. NeuroImage 2021; 237: 118174. 34000406 10.1016/j.neuroimage.2021.118174

[18] Iglesias JE, Augustinack JC, Nguyen K, Player CM, Player A, Wright M, et al. A computational atlas of the hippocampal formation using ex vivo, ultra-high resolution MRI: application to adaptive segmentation of in vivo MRI. Neuroimage 2015; 115: 117–37. 25936807 10.1016/j.neuroimage.2015.04.042 PMC4461537

[19] Kroenke K, Spitzer RL, Williams JB. The PHQ-9: validity of a brief depression severity measure. J Gen Intern Med 2001; 16: 606–13. 11556941 10.1046/j.1525-1497.2001.016009606.x PMC1495268

[20] American Psychiatric Association. DSM-IV: Diagnostic and Statistical Manual of Mental Disorders. American Psychiatric Association, 1994.

[21] Pettersson A, Boström KB, Gustavsson P, Ekselius L. Which instruments to support diagnosis of depression have sufficient accuracy? a systematic review. Nord J Psychiatry 2015; 69: 497–508. 25736983 10.3109/08039488.2015.1008568

[22] Janssen EP, Köhler S, Stehouwer CD, Schaper NC, Dagnelie PC, Sep SJ, et al. The patient health questionnaire-9 as a screening tool for depression in individuals with type 2 diabetes mellitus: the Maastricht study. J Am Geriatr Soc 2016; 64: e201–6. 27783384 10.1111/jgs.14388

[23] World Health Organization. Global Report on Diabetes: Executive Summary. WHO, 2016.

[24] Nyholt DR. A simple correction for multiple testing for single-nucleotide polymorphisms in linkage disequilibrium with each other. Am J Hum Gen 2004; 74: 765–9. 10.1086/383251 PMC1181954 14997420

[25] Sheehan DV, Lecrubier Y, Sheehan KH, Amorim P, Janavs J, Weiller E, et al. The Mini-International Neuropsychiatric Interview (MINI): the development and validation of a structured diagnostic psychiatric interview for DSM-IV and ICD-10. J Clin Psychiatry 1998; 59(Suppl 20): 22–33; quiz 4–57. 9881538

[26] Folstein MF, Folstein SE, McHugh PR. ‘Mini-mental state’: a practical method for grading the cognitive state of patients for the clinician. J Psychiatr Res 1975; 12(3): 189–98. 1202204 10.1016/0022-3956(75)90026-6

[27] Hu X, Zhang L, Hu X, Lu L, Tang S, Li H, et al. Abnormal hippocampal subfields may be potential predictors of worse early response to antidepressant treatment in drug-naïve patients with major depressive disorder. J Magn Reson Imaging 2019; 49: 1760–8. 30295348 10.1002/jmri.26520

[28] Han K-M, Won E, Sim Y, Tae W-S. Hippocampal subfield analysis in medication-naive female patients with major depressive disorder. J Affect Disord 2016; 194: 21–9. 26802503 10.1016/j.jad.2016.01.019

[29] Muñoz-Navarro R, Cano-Vindel A, Medrano LA, Schmitz F, Ruiz-Rodríguez P, Abellán-Maeso C, et al. Utility of the PHQ-9 to identify major depressive disorder in adult patients in Spanish primary care centres. BMC Psychiatry 2017; 17: 1–9. 28793892 10.1186/s12888-017-1450-8 PMC5550940

[30] Kraus C, Seiger R, Pfabigan DM, Sladky R, Tik M, Paul K, et al. Hippocampal subfields in acute and remitted depression – an ultra-high field magnetic resonance imaging study. Int J Neuropsychopharmacol 2019; 22: 513–22. 31175352 10.1093/ijnp/pyz030 PMC6672627

[31] Cao B, Luo Q, Fu Y, Du L, Qiu T, Yang X, et al. Predicting individual responses to the electroconvulsive therapy with hippocampal subfield volumes in major depression disorder. Sci Rep 2018; 8: 1–8. 29615675 10.1038/s41598-018-23685-9 PMC5882798

[32] Stockmeier CA, Mahajan GJ, Konick LC, Overholser JC, Jurjus GJ, Meltzer HY, et al. Cellular changes in the postmortem hippocampus in major depression. Biol Psychiatry 2004; 56: 640–50. 15522247 10.1016/j.biopsych.2004.08.022 PMC2929806

[33] Nestler EJ, Barrot M, DiLeone RJ, Eisch AJ, Gold SJ, Monteggia LM. Neurobiology of depression. Neuron 2002; 34: 13–25. 11931738 10.1016/s0896-6273(02)00653-0

[34] Martin A, Rief W, Klaiberg A, Braehler E. Validity of the brief patient health questionnaire mood scale (PHQ-9) in the general population. Gen Hosp Psychiatry 2006; 28: 71–7. 16377369 10.1016/j.genhosppsych.2005.07.003

[35] Negeri ZF, Levis B, Sun Y, He C, Krishnan A, Wu Y, et al. Accuracy of the patient health questionnaire-9 for screening to detect major depression: updated systematic review and individual participant data meta-analysis. Br Med J 2021; 375: n2183. 34610915 10.1136/bmj.n2183 PMC8491108

[36] Siddiqui S. Depression in type 2 diabetes mellitus—a brief review. Diabetes Metab Syndr 2014; 8: 62–5. 24661762 10.1016/j.dsx.2013.06.010

[37] Tae WS, Kim SS, Lee KU, Nam E-C, Kim KW. Validation of hippocampal volumes measured using a manual method and two automated methods (FreeSurfer and IBASPM) in chronic major depressive disorder. Neuroradiology 2008; 50: 569. 18414838 10.1007/s00234-008-0383-9

[38] Brown EM, Pierce ME, Clark DC, Fischl BR, Iglesias JE, Milberg WP, et al. Test-retest reliability of FreeSurfer automated hippocampal subfield segmentation within and across scanners. Neuroimage 2020; 210: 116563. 31972281 10.1016/j.neuroimage.2020.116563

[39] Quattrini G, Pievani M, Jovicich J, Aiello M, Bargalló N, Barkhof F, et al. Amygdalar nuclei and hippocampal subfields on MRI: test-retest reliability of automated volumetry across different MRI sites and vendors. NeuroImage 2020; 218: 116932. 32416226 10.1016/j.neuroimage.2020.116932

[40] Sämann PG, Iglesias JE, Gutman B, Grotegerd D, Leenings R, Flint C, et al. Freesurfer-based segmentation of hippocampal subfields: a review of methods and applications, with a novel quality control procedure for ENIGMA studies and other collaborative efforts. Hum Brain Mapp 2018; 43: 207–33. 10.1002/hbm.25326 PMC8805696 33368865

[41] Seiger R, Hammerle FP, Godbersen GM, Reed MB, Spurny-Dworak B, Handschuh P, et al. Comparison and reliability of hippocampal subfield segmentations within FreeSurfer utilizing T1- and T2-weighted multispectral MRI data. Front Neurosci 2021; 15: 666000. 34602964 10.3389/fnins.2021.666000 PMC8480394

