# Supplementary material for: Association of hippocampal subfield volumes with prevalence, course and incidence of depressive symptoms: The Maastricht Study
Source: Br J Psychiatry. 2024 Feb;224(2):66–73. doi: 10.1192/bjp.2023.143 (PMC10807974; doi:10.1192/bjp.2023.143)
Supplement: Monereo-Sánchez et al. supplementary material 1 — Monereo-Sánchez et al. supplementary material [file S0007125023001435sup001.docx]

# Supplements

- Supplementary Figure S1: Flowchart of the study population
- Supplementary Table S1: Hippocampal subfields description
- Supplementary Table S2: Hippocampal subfields correlation matrix
- Supplementary Figure S2: Hippocampal subfields map legend
- Supplementary Table S3: Characteristics of excluded vs. included participants
- Supplementary Table S4: Results, prevalent depressive symptoms
- Supplementary Figure S3: Hippocampal map, prevalent depressive symptoms
- Supplementary Table S5: Results, prevalent clinically relevant depressive symptoms
- Supplementary Table S6: Results, course of prevalent depression
- Supplementary Table S7: Results, incident clinically relevant depressive symptoms
- Supplementary Table S8: Results, course of incident depression
- Supplementary Table S9: Sensitivity analysis, prevalent depressive symptoms
- Supplementary Table 10: Sensitivity analysis, prevalent clinically relevant depressive symptoms
- Supplementary Table S11: Sensitivity analysis, chronic depressive symptoms

***Supplementary Figure S1|*** *Flowchart of the study population*

*
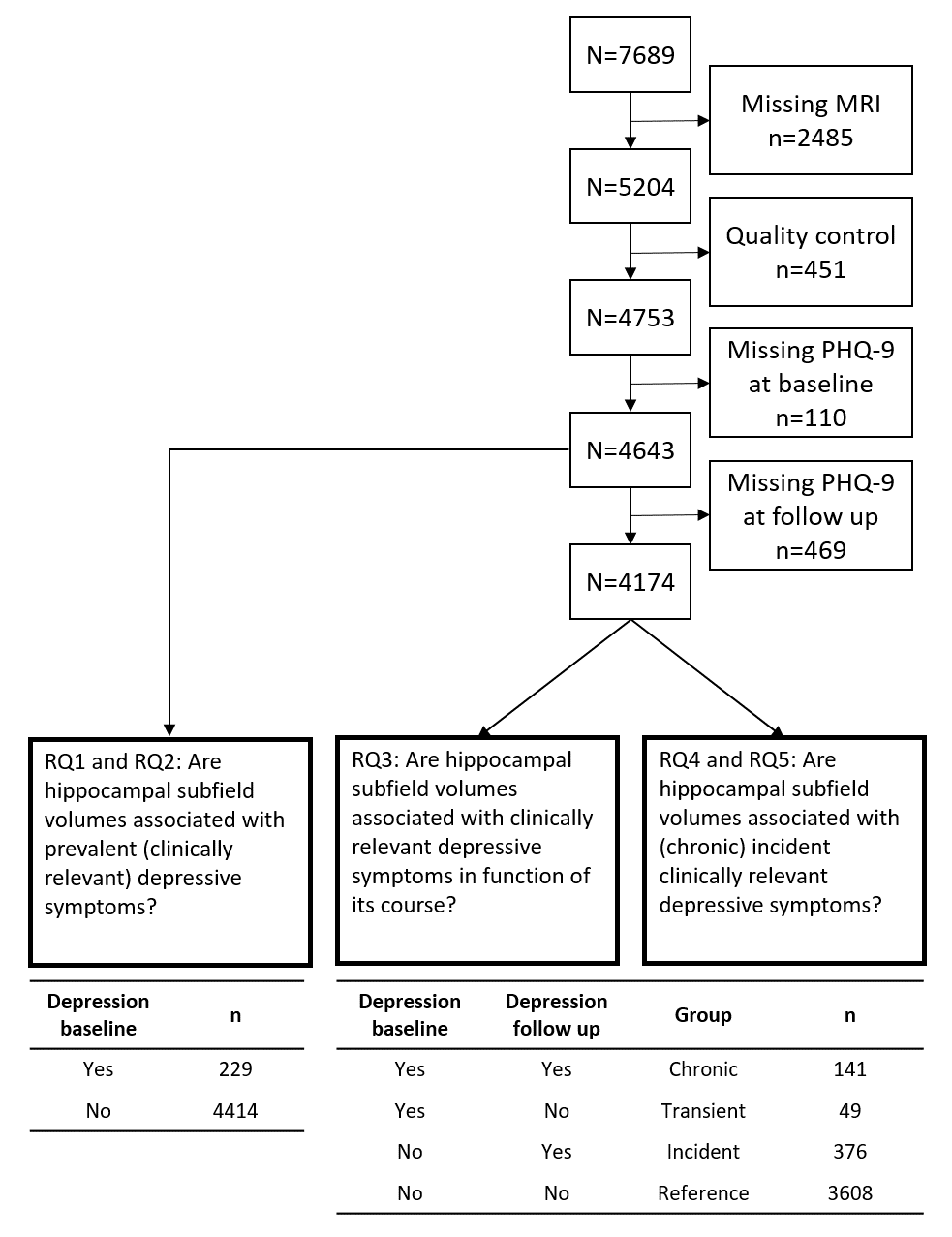
*

*From the initial 7685 participants, baseline magnetic resonance imaging (MRI) data were available from n=5,204 participants. Quality control was then applied to the MRI data through the exclusion of outliers based on Euler Numbers, following guidelines ^1^. Further, we excluded individuals with missing PHQ-9 data at baseline, resulting in a cross sectional study population of n=4643.With this sample we answered the research questions (RQ) 1 and 2 We then excluded participants with missing follow up data, resulting in a longitudinal sample of n=4174 participants. This sample is then divided by clinically relevant depressive symptoms at baseline and follow up to answer RQ3 and 4. Chronic depression contains those with depression at baseline and follow up, transient depression contains those with depression at baseline but not at follow up. Incident depression contains participants with no depression at baseline but at least one episode during follow up. The reference group contains participants with no depression at baseline nor at follow up. Subsequently, missing data among the covariates was excluded. Hence, Model 2 uses 76 less cases than Model 1 due to missing data in: education level (n=47), history of cardiovascular diseases (n=42), smoking and alcohol intake (n=25), cholesterol ratio (n=3), and waist circumference (n=2). Overall, during 18,328 person-years of follow-up, 517 (2.8%) participants developed clinically relevant depressive symptoms during follow-up. Data was available among 88.0% (year 1), 79.6% (year 2), 74.0% (year 3), 65.4% (year 4), 57.9% (year 5), 35.8% (year 6), and 19.1% (year 7) of the participants. It is important to note that the lower percentages after the fifth year are a result of the ongoing annual follow-up from year 6 onwards.*

***Supplementary Table S1 |*** *Hippocampal subfields description*

| In text name | Description (FreeSurfer name) | Left volume mm^3^  (mean ± SD) | Right volume mm^3^  (mean ± SD) |
| --- | --- | --- | --- |
| Hippocampus total volume | Total Hippocampal Volume (Whole_hippocampus) | 3414.18 ± 354.78 | 3436.48 ± 356.99 |
| Tail | Hippocampal tail (Hippocampal_tail) | 533.86 ± 73.08 | 509.64 ± 68.47 |
| Subiculum | Subiculum (Subiculum) | 470.50 ± 53.94 | 468.95 ± 52.34 |
| CA1 | Cornu Ammonis 1 (CA1) | 709.24 ± 86.80 | 740.29 ± 91.22 |
| Fissure | Hippocampal fissure (Hippocampal-fissure) | 77.85 ± 15.44 | 84.93 ± 17.22 |
| Presubiculum | Presubiculum (Presubiculum) | 307.61 ± 40.18 | 290.93 ± 38.54 |
| Parasubiculum | Parasubiculum (Parasubiculum) | 61.63 ± 13.12 | 61.19 ± 12.51 |
| Molecular layer | Molecular layer of the hippocampus (Molecular_layer_HP) | 351.56 ± 49.56 | 371.96 ± 49.80 |
| Dentate gyrus | Granule cell and molecular cell layer of the dentate gyrus (GC_ML_DG) | 333.98 ± 40.38 | 333.96 ± 40.84 |
| CA3 | Cornu Ammonis 2 and 3 (CA3) | 231.63 ± 39.10 | 248.77 ± 39.84 |
| CA4 | Cornu Ammonis 4 (CA4) | 273.66 ± 30.90 | 276.85 ± 32.03 |
| Fimbria | Fimbria (Fimbria) | 79.63 ± 23.69 | 71.12 ± 23.18 |
| HATA | Hippocampus-amygdala-transition-area (HATA) | 60.87 ± 10.88 | 62.83 ± 10.42 |

*Table shows the abbreviation and complete name, as well as the mean volume (mm^3^)* ± *standard deviation (SD) for total hippocampal volume and each hippocampal subfield of the cross sectional sample.*

***Supplementary Table S2 |*** *Hippocampal subfields correlation matrix*

|  | **LH Tail** | **LH Subiculum** | **LH CA1** | **LH Fissure** | **LH Presubiculum** | **LH Parasubiculum** | **LH Molecular layer** | **LH Dentate gyrus** | **LH CA3** | **LH CA4** | **LH Fimbria** | **LH HATA** | **RH Tail** | **RH Subiculum** | **RH CA1** | **RH Fissure** | **RH Presubiculum** | **RH Parasubiculum** | **RH Molecular layer** | **RH Dentate gyrus** | **RH CA3** | **RH CA4** | **RH Fimbria** | **RH HATA** |
| --- | --- | --- | --- | --- | --- | --- | --- | --- | --- | --- | --- | --- | --- | --- | --- | --- | --- | --- | --- | --- | --- | --- | --- | --- |
| **LH Tail** | 1 | 0.53 | 0.58 | 0.21 | 0.43 | 0.25 | 0.37 | 0.53 | 0.53 | 0.57 | 0.3 | 0.32 | 0.82 | 0.59 | 0.57 | 0.2 | 0.48 | 0.24 | 0.41 | 0.52 | 0.45 | 0.52 | 0.32 | 0.34 |
| **LH Subiculum** | 0.53 | 1 | 0.69 | 0.33 | 0.76 | 0.38 | 0.6 | 0.69 | 0.3 | 0.68 | 0.39 | 0.49 | 0.54 | 0.82 | 0.67 | 0.27 | 0.65 | 0.34 | 0.54 | 0.61 | 0.35 | 0.59 | 0.37 | 0.48 |
| **LH CA1** | 0.58 | 0.69 | 1 | 0.44 | 0.49 | 0.32 | 0.66 | 0.78 | 0.67 | 0.81 | 0.45 | 0.56 | 0.59 | 0.68 | 0.83 | 0.33 | 0.52 | 0.3 | 0.61 | 0.74 | 0.59 | 0.73 | 0.42 | 0.54 |
| **LH Fissure** | 0.21 | 0.33 | 0.44 | 1 | 0.12 | -0.04 | 0.64 | 0.2 | 0.26 | 0.33 | -0.07 | 0.16 | 0.22 | 0.33 | 0.41 | 0.63 | 0.16 | 0.04 | 0.51 | 0.28 | 0.19 | 0.34 | -0.01 | 0.2 |
| **LH Presubiculum** | 0.43 | 0.76 | 0.49 | 0.12 | 1 | 0.53 | 0.44 | 0.59 | 0.19 | 0.53 | 0.42 | 0.49 | 0.45 | 0.66 | 0.48 | 0.12 | 0.74 | 0.38 | 0.38 | 0.48 | 0.27 | 0.45 | 0.36 | 0.44 |
| **LH Parasubiculum** | 0.25 | 0.38 | 0.32 | -0.04 | 0.53 | 1 | 0.31 | 0.42 | 0.18 | 0.39 | 0.23 | 0.38 | 0.24 | 0.32 | 0.27 | 0.03 | 0.38 | 0.52 | 0.24 | 0.35 | 0.21 | 0.33 | 0.21 | 0.35 |
| **LH Molecular layer** | 0.37 | 0.6 | 0.66 | 0.64 | 0.44 | 0.31 | 1 | 0.53 | 0.34 | 0.61 | 0.18 | 0.39 | 0.38 | 0.53 | 0.59 | 0.47 | 0.38 | 0.27 | 0.73 | 0.52 | 0.32 | 0.55 | 0.19 | 0.41 |
| **LH Dentate gyrus** | 0.53 | 0.69 | 0.78 | 0.2 | 0.59 | 0.42 | 0.53 | 1 | 0.66 | 0.96 | 0.55 | 0.63 | 0.54 | 0.64 | 0.71 | 0.18 | 0.55 | 0.37 | 0.5 | 0.83 | 0.62 | 0.8 | 0.47 | 0.58 |
| **LH CA3** | 0.53 | 0.3 | 0.67 | 0.26 | 0.19 | 0.18 | 0.34 | 0.66 | 1 | 0.74 | 0.3 | 0.39 | 0.5 | 0.39 | 0.57 | 0.18 | 0.3 | 0.19 | 0.36 | 0.63 | 0.71 | 0.64 | 0.31 | 0.38 |
| **LH CA4** | 0.57 | 0.68 | 0.81 | 0.33 | 0.53 | 0.39 | 0.61 | 0.96 | 0.74 | 1 | 0.43 | 0.57 | 0.57 | 0.66 | 0.74 | 0.28 | 0.53 | 0.35 | 0.57 | 0.83 | 0.64 | 0.82 | 0.4 | 0.56 |
| **LH Fimbria** | 0.3 | 0.39 | 0.45 | -0.07 | 0.42 | 0.23 | 0.18 | 0.55 | 0.3 | 0.43 | 1 | 0.51 | 0.34 | 0.36 | 0.44 | -0.08 | 0.35 | 0.22 | 0.22 | 0.5 | 0.41 | 0.42 | 0.7 | 0.43 |
| **LH HATA** | 0.32 | 0.49 | 0.56 | 0.16 | 0.49 | 0.38 | 0.39 | 0.63 | 0.39 | 0.57 | 0.51 | 1 | 0.35 | 0.45 | 0.53 | 0.12 | 0.44 | 0.34 | 0.36 | 0.56 | 0.44 | 0.53 | 0.42 | 0.63 |
| **RH Tail** | 0.82 | 0.54 | 0.59 | 0.22 | 0.45 | 0.24 | 0.38 | 0.54 | 0.5 | 0.57 | 0.34 | 0.35 | 1 | 0.62 | 0.62 | 0.23 | 0.51 | 0.27 | 0.45 | 0.56 | 0.5 | 0.57 | 0.34 | 0.37 |
| **RH Subiculum** | 0.59 | 0.82 | 0.68 | 0.33 | 0.66 | 0.32 | 0.53 | 0.64 | 0.39 | 0.66 | 0.36 | 0.45 | 0.62 | 1 | 0.73 | 0.34 | 0.77 | 0.36 | 0.62 | 0.62 | 0.35 | 0.62 | 0.38 | 0.47 |
| **RH CA1** | 0.57 | 0.67 | 0.83 | 0.41 | 0.48 | 0.27 | 0.59 | 0.71 | 0.57 | 0.74 | 0.44 | 0.53 | 0.62 | 0.73 | 1 | 0.46 | 0.48 | 0.28 | 0.74 | 0.78 | 0.64 | 0.78 | 0.4 | 0.57 |
| **RH Fissure** | 0.2 | 0.27 | 0.33 | 0.63 | 0.12 | 0.03 | 0.47 | 0.18 | 0.18 | 0.28 | -0.08 | 0.12 | 0.23 | 0.34 | 0.46 | 1 | 0.07 | -0.02 | 0.63 | 0.23 | 0.23 | 0.33 | -0.13 | 0.18 |
| **RH Presubiculum** | 0.48 | 0.65 | 0.52 | 0.16 | 0.74 | 0.38 | 0.38 | 0.55 | 0.3 | 0.53 | 0.35 | 0.44 | 0.51 | 0.77 | 0.48 | 0.07 | 1 | 0.48 | 0.38 | 0.49 | 0.24 | 0.45 | 0.41 | 0.45 |
| **RH Parasubiculum** | 0.24 | 0.34 | 0.3 | 0.04 | 0.38 | 0.52 | 0.27 | 0.37 | 0.19 | 0.35 | 0.22 | 0.34 | 0.27 | 0.36 | 0.28 | -0.02 | 0.48 | 1 | 0.27 | 0.33 | 0.17 | 0.31 | 0.25 | 0.44 |
| **RH Molecular layer** | 0.41 | 0.54 | 0.61 | 0.51 | 0.38 | 0.24 | 0.73 | 0.5 | 0.36 | 0.57 | 0.22 | 0.36 | 0.45 | 0.62 | 0.74 | 0.63 | 0.38 | 0.27 | 1 | 0.59 | 0.41 | 0.65 | 0.17 | 0.44 |
| **RH Dentate gyrus** | 0.52 | 0.61 | 0.74 | 0.28 | 0.48 | 0.35 | 0.52 | 0.83 | 0.63 | 0.83 | 0.5 | 0.56 | 0.56 | 0.62 | 0.78 | 0.23 | 0.49 | 0.33 | 0.59 | 1 | 0.77 | 0.98 | 0.45 | 0.6 |
| **RH CA3** | 0.45 | 0.35 | 0.59 | 0.19 | 0.27 | 0.21 | 0.32 | 0.62 | 0.71 | 0.64 | 0.41 | 0.44 | 0.5 | 0.35 | 0.64 | 0.23 | 0.24 | 0.17 | 0.41 | 0.77 | 1 | 0.8 | 0.32 | 0.45 |
| **RH CA4** | 0.52 | 0.59 | 0.73 | 0.34 | 0.45 | 0.33 | 0.55 | 0.8 | 0.64 | 0.82 | 0.42 | 0.53 | 0.57 | 0.62 | 0.78 | 0.33 | 0.45 | 0.31 | 0.65 | 0.98 | 0.8 | 1 | 0.36 | 0.57 |
| **RH Fimbria** | 0.32 | 0.37 | 0.42 | -0.01 | 0.36 | 0.21 | 0.19 | 0.47 | 0.31 | 0.4 | 0.7 | 0.42 | 0.34 | 0.38 | 0.4 | -0.13 | 0.41 | 0.25 | 0.17 | 0.45 | 0.32 | 0.36 | 1 | 0.45 |
| **RH HATA** | 0.34 | 0.48 | 0.54 | 0.2 | 0.44 | 0.35 | 0.41 | 0.58 | 0.38 | 0.56 | 0.43 | 0.63 | 0.37 | 0.47 | 0.57 | 0.18 | 0.45 | 0.44 | 0.44 | 0.6 | 0.45 | 0.57 | 0.45 | 1 |

*Table shows Pearson correlation coefficients between each pairwise combination of hippocampal subfields. Color intensity indicates correlation strength. Abbreviations: LH, left hemisphere; RH, right hemisphere; CA, Cornu Ammonis; HATA, Hippocampus-amygdala-transition-area.*

***Supplementary Figure S2|*** *Hippocampal subfields map legend*


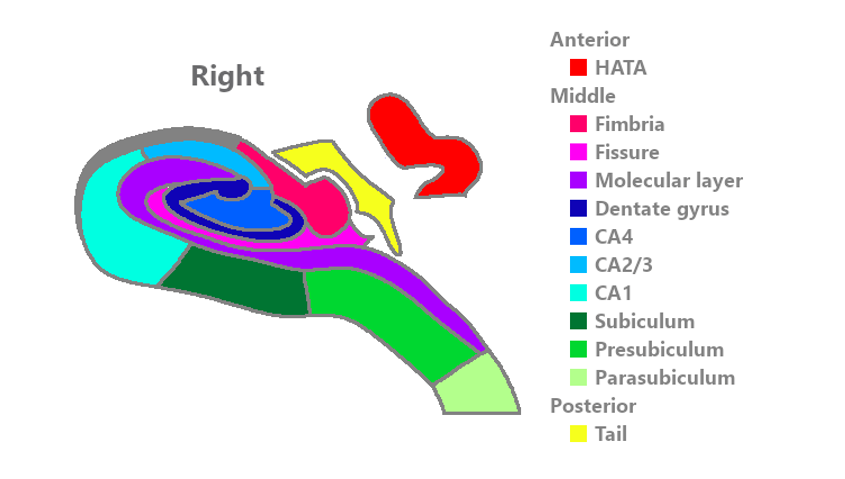


*Figure shows a schematic representation of the right hippocampus in a coronal section. Hippocampal subfields are represented in different colors. Notice than the subfield HATA is transposed from an anterior coronal section, while Tail is transposed from a posterior coronal section. Abbreviations: HATA, Hippocampus-amygdala-transition-area; CA, Cornu Ammonis.*

***Supplementary Table S3 |*** *Characteristics of excluded vs. included participants*

| Characteristic | **Excluded**  **n=3046** | **Included**  **n=4643** | **p value** |
| --- | --- | --- | --- |
| Age | 66 ± 8 | 60 ± 8.6 | **p<0.001** |
| Sex (% female) | 46.4 | 51.8 | **p<0.001** |
| BMI (Km/cm^3^) | 28 ± 5 | 26.4 ± 4.1 | **p<0.001** |
| Waist (cm) | 98.8 ± 14.6 | 93 ± 12.6 | **p<0.001** |
| Education  (% Low, Medium, High) | 41.3, 26.1, 32.6 | 30.4, 28.6, 40.9 | **p<0.001** |
| Alcohol consumption  (% None, Low, High) | 21.2, 56.8, 22 | 16.8, 59, 24.3 | **p<0.001** |
| Smoking status  (% Never, Former, Current) | 32.9, 51.4, 15.7 | 40, 48.1, 12 | **p<0.001** |
| Partner (% yes) | 80.6 | 85 | **p<0.001** |
| T2DM (% yes)^α^ | 34.6 | 18.1 | **p<0.001** |
| Cardiovascular disease (% yes) | 26.1 | 11.2 | **p<0.001** |
| Hypertension (% yes) | 64.4 | 47 | **p<0.001** |
| Cholesterol ratio | 3.7 ± 1.2 | 3.6 ± 1.2 | **p<0.001** |
| Cholesterol medication (% yes) | 43.4 | 25.5 | **p<0.001** |
| Antidepressants (% yes) | 8.1 | 6.6 | **p=0,015** |
| History of depression (% yes) | 33.5 | 30.1 | **p=0,0018** |

*Table shows the demographics and group comparison results between included and excluded participants. Data is presented as mean ± SD or percentage as appropriate. Participants not included in this study due to unavailable MRI (n=2485), low quality MRI (n=451), or missing baseline PHQ-9 data (n=110) were statistically significantly older, more often men, had a higher waist circumference, had lower education level, had a higher cardiovascular risk profile, and were more prone to take antidepressants than participants included in the analyses. Abbreviations: PHQ-9, patient health questionnaire; BMI, Body Mass Index; T2DM, Type 2 diabetes mellitus; CVD, cardiovascular disease. Bold shows p<0.05.*

*^α^ Study design is oversampled with individuals with type 2 diabetes for reasons of efficiency.*

***Supplementary Table S4|*** *Results, prevalent depressive symptoms (PHQ-9 score)*

|  |  | Model 1 |  |  | Model 2 |  |  |
| --- | --- | --- | --- | --- | --- | --- | --- |
|  | **Structure** | **RR(95%CI)** | **p value** |  | **RR(95%CI)** | **p value** |  |
| Left hemisphere | | | | | | | |
|  | Total Hippocampus volume | 0.98(0.93;1.03) | 0.427 |  | 0.98(0.94;1.03) | 0.477 |  |
|  | *Subfields* |  |  |  |  |  |  |
|  | Tail | 1.03(0.98;1.08) | 0.288 |  | 1.02(0.97;1.07) | 0.441 |  |
|  | Subiculum | 1.03(0.97;1.10) | 0.305 |  | 1.03(0.97;1.10) | 0.279 |  |
|  | Cornu Ammonis 1 (CA1) | 0.99(0.92;1.08) | 0.901 |  | 1.01(0.93;1.09) | 0.779 |  |
|  | Fissure | **1.05(1.01;1.09)** | **0.013** |  | 1.04(1.00;1.08) | 0.054 |  |
|  | Presubiculum | 0.98(0.93;1.02) | 0.309 |  | 0.98(0.94;1.03) | 0.526 |  |
|  | Parasubiculum | 0.97(0.94;1.01) | 0.175 |  | 0.98(0.94;1.02) | 0.287 |  |
|  | Molecular layer | **1.11(1.05;1.17)** | **p<0.001** | ***** | **1.06(1.01;1.12)** | **0.029** |  |
|  | Dentate gyrus | **0.92(0.85;0.99)** | **0.021** |  | **0.93(0.86;1.00)** | **0.040** |  |
|  | Cornu Ammonis 3 (CA3) | **0.94(0.90;0.98)** | **0.009** |  | 0.96(0.91;1.00) | 0.062 |  |
|  | Cornu Ammonis 4 (CA4) | 0.95(0.88;1.03) | 0.221 |  | 0.96(0.88;1.04) | 0.261 |  |
|  | Fimbria | 0.98(0.94;1.02) | 0.390 |  | 0.98(0.94;1.03) | 0.436 |  |
|  | HATA | 0.99(0.95;1.04) | 0.756 |  | 1.00(0.96;1.05) | 0.917 |  |
| Right hemisphere | | | | | | | |
|  | Total Hippocampus volume | 0.97(0.92;1.02) | 0.214 |  | 0.97(0.92;1.01) | 0.170 |  |
|  | *Subfields* |  |  |  |  |  |  |
|  | Tail | 0.99(0.94;1.04) | 0.649 |  | 0.99(0.94;1.05) | 0.847 |  |
|  | subiculum | 1.03(0.97;1.10) | 0.304 |  | 1.05(0.99;1.12) | 0.133 |  |
|  | Cornu Ammonis 1 (CA1) | **1.09(1.01;1.19)** | **0.040** |  | 1.07(0.98;1.16) | 0.120 |  |
|  | Fissure | **1.06(1.02;1.11)** | **0.002** | ***** | **1.04(1.00;1.08)** | **0.040** |  |
|  | Presubiculum | 0.99(0.95;1.04) | 0.744 |  | 1.00(0.96;1.05) | 0.899 |  |
|  | Parasubiculum | 1.00(0.96;1.04) | 0.955 |  | 1.00(0.96;1.04) | 0.976 |  |
|  | Molecular layer | **1.14(1.08;1.20)** | **p<0.001** | ***** | **1.08(1.02;1.14)** | **0.006** |  |
|  | Dentate gyrus | **0.89(0.83;0.96)** | **0.002** | ***** | **0.90(0.84;0.97)** | **0.005** |  |
|  | Cornu Ammonis 3 (CA3) | **0.91(0.87;0.96)** | **p<0.001** | ***** | **0.94(0.89;0.98)** | **0.007** |  |
|  | Cornu Ammonis 4 (CA4) | **0.89(0.83;0.96)** | **0.002** | ***** | **0.89(0.83;0.96)** | **0.002** | ***** |
|  | Fimbria | 0.99(0.95;1.04) | 0.799 |  | 1.00(0.96;1.04) | 0.898 |  |
|  | HATA | 0.99(0.95;1.04) | 0.821 |  | 1.00(0.95;1.04) | 0.939 |  |

*Table shows the results for negative binomial regression on depressive symptoms load. Results are presented in rate ratio (RR) and confidence intervals (CI). Bold shows nominally significant (p<0.05); Star (*) indicate a significant result after multiple comparison correction (p<0.0039). Abbreviations; HATA, Hippocampus-amygdala-transition-area. Model 1 was, adjusted for brain total volume when analyzing hippocampal total volumes, or adjusted for left/right hippocampus total volume, when analyzing hippocampal subfields, MRI lag time, age and sex; Model 2 was additionally adjusted for potential confounders: T2DM status, education level, waist circumference, history of cardiovascular disease, cholesterol ratio, use of alcohol and smoking status.*

**Supplementary Figure S3|** Hippocampal map: Prevalent depressive symptoms *(PHQ-9 score)*


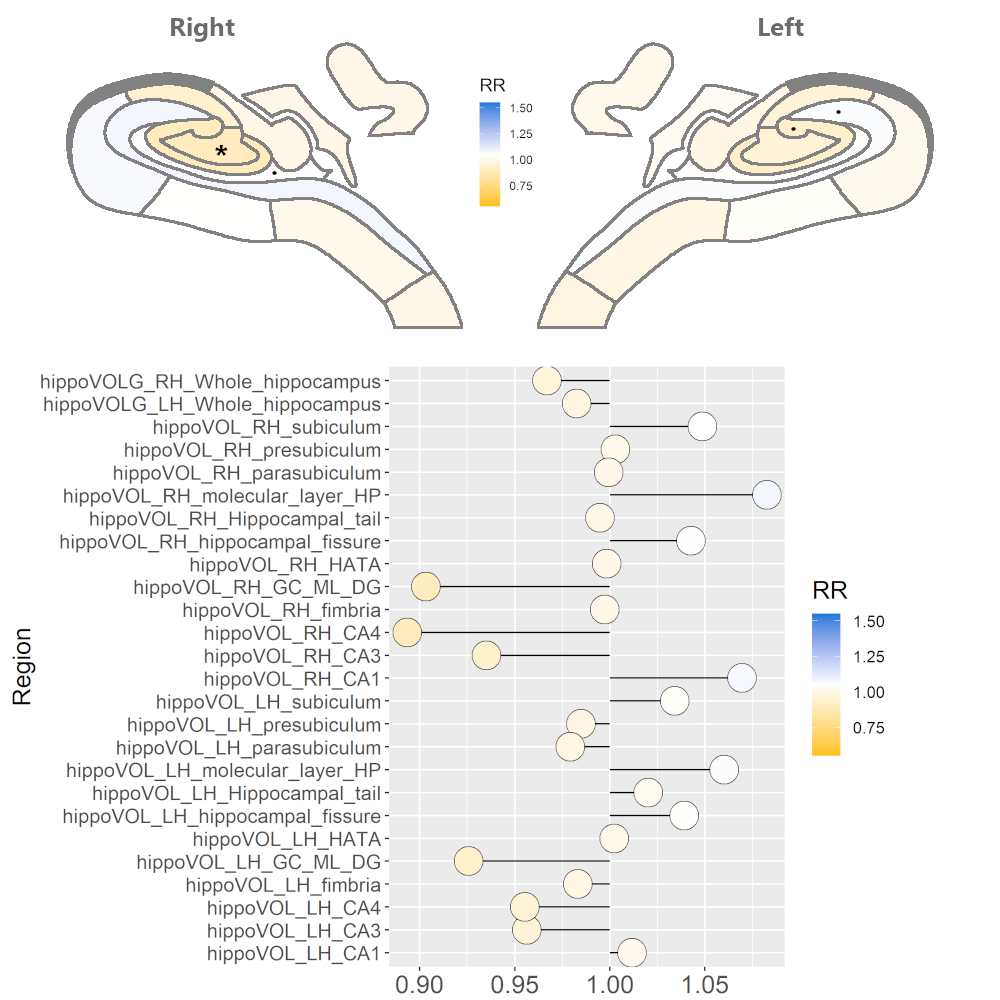


CA4

Fissure

DG

ML

.

ML

DG

.

*The hippocampal map shows the associations between hippocampal subfield volumes and prevalent depressive symptoms on a continuous scale after full adjustment (Model 2). Blue color represents a positive association: a higher volume is associated with higher rate ratio (RR) for depression, while yellow represent a negative association. Dots show the hippocampal subfields with associations of p<0.05, stars show the subfields that are significant after multiple comparison correction (p<0.0039). See Supplementary figure S2 for a hippocampal map legend. Abbreviations: CA, Cornu Ammonis; DG, Dentate gyrus; ML, Molecular layer.*

***Supplementary Table S5|*** *Results, prevalent clinically relevant depressive symptoms (PHQ-9≥10)*

|  |  | Model 1 |  |  | Model 2 |  |  |
| --- | --- | --- | --- | --- | --- | --- | --- |
|  | **Structure** | **OR(95%CI)** | **p value** |  | **OR(95%CI)** | **p value** |  |
| Left hemisphere | | | | | | |  |
|  | Total Hippocampus volume | 0.97(0.79;1.17) | 0.723 |  | 0.96(0.78;1.17) | 0.659 |  |
|  | *Subfields* |  |  |  |  |  |  |
|  | Tail | **1.25(1.02;1.53)** | **0.032** |  | 1.22(0.99;1.50) | 0.063 |  |
|  | Subiculum | 1.16(0.91;1.47) | 0.236 |  | 1.18(0.92;1.52) | 0.187 |  |
|  | Cornu Ammonis 1 (CA1) | 0.92(0.67;1.28) | 0.624 |  | 0.94(0.67;1.31) | 0.704 |  |
|  | Fissure | **1.23(1.06;1.42)** | **0.006** |  | **1.20(1.03;1.39)** | **0.017** |  |
|  | Presubiculum | 0.90(0.74;1.09) | 0.290 |  | 0.92(0.75;1.11) | 0.376 |  |
|  | Parasubiculum | **0.82(0.70;0.96)** | **0.012** |  | **0.82(0.70;0.96)** | **0.014** |  |
|  | Molecular layer | **1.27(1.04;1.55)** | **0.018** |  | 1.17(0.95;1.44) | 0.146 |  |
|  | Dentate gyrus | **0.71(0.53;0.96)** | **0.025** |  | **0.74(0.54;1.00)** | **0.048** |  |
|  | Cornu Ammonis 3 (CA3) | 0.86(0.71;1.03) | 0.107 |  | 0.89(0.73;1.09) | 0.262 |  |
|  | Cornu Ammonis 4 (CA4) | 0.85(0.61;1.17) | 0.321 |  | 0.87(0.63;1.22) | 0.425 |  |
|  | Fimbria | 0.90(0.76;1.07) | 0.221 |  | 0.91(0.76;1.09) | 0.323 |  |
|  | HATA | 0.85(0.71;1.01) | 0.068 |  | 0.86(0.72;1.03) | 0.110 |  |
| Right hemisphere | | | | | | |  |
|  | Total Hippocampus volume | 0.93(0.76;1.13) | 0.455 |  | 0.90(0.74;1.10) | 0.318 |  |
|  | *Subfields* |  |  |  |  |  |  |
|  | Tail | 1.05(0.85;1.30) | 0.646 |  | 1.04(0.83;1.29) | 0.751 |  |
|  | subiculum | **1.30(1.01;1.67)** | **0.039** |  | **1.38(1.07;1.78)** | **0.014** |  |
|  | Cornu Ammonis 1 (CA1) | 1.31(0.93;1.85) | 0.125 |  | 1.29(0.91;1.83) | 0.154 |  |
|  | Fissure | **1.32(1.13;1.53)** | **p<0.001** | ***** | **1.26(1.08;1.48)** | **0.003** | ***** |
|  | Presubiculum | 0.91(0.76;1.10) | 0.342 |  | 0.94(0.78;1.14) | 0.518 |  |
|  | Parasubiculum | 0.93(0.80;1.08) | 0.353 |  | 0.93(0.79;1.09) | 0.357 |  |
|  | Molecular layer | **1.46(1.18;1.81)** | **p<0.001** | ***** | **1.31(1.05;1.64)** | **0.015** |  |
|  | Dentate gyrus | **0.69(0.51;0.92)** | **0.012** |  | **0.69(0.51;0.93)** | **0.014** |  |
|  | Cornu Ammonis 3 (CA3) | **0.72(0.59;0.87)** | **p<0.001** | ***** | **0.75(0.62;0.92)** | **0.006** |  |
|  | Cornu Ammonis 4 (CA4) | **0.70(0.52;0.94)** | **0.019** |  | **0.68(0.50;0.92)** | **0.013** |  |
|  | Fimbria | 0.93(0.79;1.10) | 0.394 |  | 0.95(0.80;1.13) | 0.583 |  |
|  | HATA | 0.91(0.77;1.09) | 0.321 |  | 0.91(0.75;1.09) | 0.287 |  |

*Table shows the results for logistic regression on prevalent clinical depression. Results are presented in odds ratio (OR) and confidence intervals (CI). Bold shows nominally significant (p<0.05); Star (*) shows multiple comparison correction significant (p<0.0039). Abbreviations; HATA, Hippocampus-amygdala-transition-area. Model 1 was, adjusted for brain total volume when analyzing hippocampal total volumes, or adjusted for left/right hippocampus total volume, when analyzing hippocampal subfields, MRI lag time, age and sex; Model 2 was additionally adjusted for potential confounders: T2DM status, education level, waist circumference, history of cardiovascular disease, cholesterol ratio, use of alcohol and smoking status.*

***Supplementary Table S6|*** *Results, course of prevalent depression (chronic or transient)*

|  |  | A. Transient course | | | | | | B. Chronic course | | | | | |
| --- | --- | --- | --- | --- | --- | --- | --- | --- | --- | --- | --- | --- | --- |
|  |  | **Model 1** |  |  | **Model 2** |  |  | **Model 1** |  |  | **Model 2** |  |  |
|  | **Structure** | **OR(95%CI)** | **P value** |  | **OR(95%CI)** | **P value** |  | **OR(95%CI)** | **P value** |  | **OR(95%CI)** | **P value** |  |
| Left hemisphere | | | | | | | | | | | | | |
|  | Total Hippocampus volume | 1.45(0.97;2.16) | 0.068 |  | 1.46(0.96;2.21) | 0.073 |  | **0.73(0.56;0.94)** | **0.013** |  | **0.70(0.54;0.91)** | **0.009** |  |
|  | *Subfields* |  |  |  |  |  |  |  |  |  |  |  |  |
|  | Tail | 1.11(0.74;1.69) | 0.608 |  | 1.09(0.71;1.66) | 0.698 |  | 1.29(0.99;1.68) | 0.060 |  | 1.24(0.95;1.63) | 0.114 |  |
|  | Subiculum | 0.95(0.58;1.56) | 0.849 |  | 0.92(0.55;1.55) | 0.759 |  | 1.10(0.80;1.51) | 0.562 |  | 1.17(0.84;1.61) | 0.354 |  |
|  | Cornu Ammonis 1 (CA1) | 1.27(0.65;2.46) | 0.481 |  | 1.22(0.62;2.40) | 0.569 |  | 0.99(0.65;1.50) | 0.948 |  | 1.04(0.68;1.60) | 0.849 |  |
|  | Fissure | 0.95(0.68;1.33) | 0.753 |  | 0.95(0.67;1.34) | 0.758 |  | **1.42(1.18;1.70)** | **p<0.001** | ***** | **1.37(1.14;1.64)** | **p<0.001** | * |
|  | Presubiculum | 0.88(0.59;1.30) | 0.512 |  | 0.81(0.54;1.22) | 0.320 |  | 0.85(0.66;1.09) | 0.208 |  | 0.86(0.67;1.11) | 0.258 |  |
|  | Parasubiculum | 0.94(0.68;1.28) | 0.680 |  | 0.90(0.65;1.25) | 0.518 |  | **0.73(0.59;0.90)** | **0.003** | * | **0.74(0.60;0.91)** | **0.005** |  |
|  | Molecular layer | 0.83(0.53;1.31) | 0.429 |  | 0.76(0.47;1.23) | 0.271 |  | **1.45(1.13;1.85)** | **0.003** | * | **1.34(1.04;1.74)** | **0.026** |  |
|  | Dentate gyrus | 0.86(0.47;1.56) | 0.612 |  | 0.91(0.49;1.70) | 0.774 |  | 0.74(0.50;1.08) | 0.120 |  | 0.75(0.51;1.12) | 0.159 |  |
|  | Cornu Ammonis 3 (CA3) | 1.05(0.72;1.53) | 0.804 |  | 1.25(0.84;1.86) | 0.281 |  | 0.83(0.65;1.06) | 0.131 |  | 0.83(0.65;1.07) | 0.158 |  |
|  | Cornu Ammonis 4 (CA4) | 0.89(0.47;1.72) | 0.735 |  | 0.97(0.49;1.89) | 0.921 |  | 0.99(0.65;1.50) | 0.953 |  | 1.02(0.67;1.58) | 0.914 |  |
|  | Fimbria | 1.15(0.81;1.62) | 0.439 |  | 1.22(0.86;1.74) | 0.270 |  | **0.77(0.62;0.97)** | **0.025** |  | **0.78(0.61;0.98)** | **0.032** |  |
|  | HATA | 0.91(0.64;1.30) | 0.605 |  | 0.90(0.62;1.30) | 0.560 |  | 0.81(0.64;1.02) | 0.071 |  | 0.81(0.64;1.04) | 0.093 |  |
| Right hemisphere | | | | | | | | | | | | | |
|  | Total Hippocampus volume | 1.26(0.85;1.88) | 0.242 |  | 1.30(0.87;1.94) | 0.206 |  | **0.70(0.54;0.90)** | **0.006** |  | **0.68(0.52;0.87)** | **0.003** | * |
|  | *Subfields* |  |  |  |  |  |  |  |  |  |  |  |  |
|  | Tail | 0.97(0.62;1.50) | 0.877 |  | 0.98(0.63;1.53) | 0.918 |  | 1.16(0.88;1.52) | 0.296 |  | 1.14(0.86;1.50) | 0.375 |  |
|  | Subiculum | 1.04(0.63;1.72) | 0.888 |  | 1.02(0.60;1.72) | 0.953 |  | 1.29(0.94;1.79) | 0.118 |  | 1.37(0.99;1.91) | 0.059 |  |
|  | Cornu Ammonis 1 (CA1) | 1.08(0.54;2.15) | 0.824 |  | 1.12(0.55;2.26) | 0.755 |  | 1.29(0.83;2.00) | 0.260 |  | 1.30(0.83;2.03) | 0.252 |  |
|  | Fissure | 1.18(0.86;1.61) | 0.318 |  | 1.14(0.82;1.58) | 0.447 |  | **1.46(1.21;1.77)** | **p<0.001** | * | **1.40(1.15;1.71)** | **p<0.001** | * |
|  | Presubiculum | 0.77(0.52;1.13) | 0.181 |  | 0.75(0.50;1.11) | 0.152 |  | 1.01(0.79;1.28) | 0.953 |  | 1.01(0.79;1.29) | 0.938 |  |
|  | Parasubiculum | 1.11(0.82;1.51) | 0.492 |  | 1.10(0.81;1.51) | 0.534 |  | 0.87(0.71;1.07) | 0.181 |  | 0.87(0.71;1.07) | 0.179 |  |
|  | Molecular layer | 1.29(0.83;2.00) | 0.258 |  | 1.18(0.75;1.86) | 0.477 |  | **1.66(1.27;2.19)** | **p<0.001** | * | **1.51(1.14;2.00)** | **0.004** | * |
|  | Dentate gyrus | 0.99(0.56;1.75) | 0.971 |  | 1.00(0.56;1.81) | 0.989 |  | **0.62(0.43;0.90)** | **0.012** |  | **0.63(0.43;0.93)** | **0.019** |  |
|  | Cornu Ammonis 3 (CA3) | 0.98(0.66;1.46) | 0.940 |  | 1.08(0.72;1.64) | 0.698 |  | **0.60(0.47;0.77)** | **p<0.001** | * | **0.61(0.48;0.79)** | **p<0.001** | * |
|  | Cornu Ammonis 4 (CA4) | 1.09(0.61;1.94) | 0.776 |  | 1.10(0.61;2.00) | 0.749 |  | **0.61(0.42;0.89)** | **0.010** |  | **0.60(0.40;0.88)** | **0.010** |  |
|  | Fimbria | 0.83(0.59;1.17) | 0.276 |  | 0.83(0.58;1.18) | 0.299 |  | 0.97(0.78;1.20) | 0.772 |  | 1.00(0.80;1.24) | 0.973 |  |
|  | HATA | 1.25(0.88;1.79) | 0.217 |  | 1.20(0.83;1.73) | 0.340 |  | **0.76(0.61;0.96)** | **0.020** |  | **0.74(0.59;0.94)** | **0.014** |  |

*Table shows the results for multinomial logistic regression analysis on prevalent depression when it presents* ***A)*** *a transient course or B****)*** *a chronic course. Reference group: No depression at baseline nor at follow up (n=3608). Results are presented in odds ratios (OR) and confidence intervals (CI). Bold shows nominally significant (p<0.05); Star (*) shows multiple comparison correction significant (p<0.0039). Abbreviations: LH/RH, Left/right hemisphere; HC, Hippocampus; CA, Cornu Ammonis; HATA, Hippocampus-amygdala-transition-area.*

***Supplementary Table S7|*** *Results, incident clinically relevant depressive symptoms*

|  |  | Model 1 | | | Model 2 | | |
| --- | --- | --- | --- | --- | --- | --- | --- |
|  | **Structure** | **HR(95%CI)** | **P value** |  | **HR(95%CI)** | **P value** |  |
| LH | Total HC | 0.96(0.83;1.12) | 0.612 |  | 0.96(0.83;1.12) | 0.644 |  |
|  | Tail | 1.15(0.99;1.35) | 0.066 |  | 1.15(0.99;1.35) | 0.074 |  |
|  | Subiculum | **0.83(0.69;1.00)** | **0.047** |  | 0.83(0.69;1.01) | 0.057 |  |
|  | CA1 | 1.14(0.89;1.45) | 0.305 |  | 1.09(0.85;1.39) | 0.483 |  |
|  | Fissure | 1.10(0.98;1.23) | 0.118 |  | 1.05(0.94;1.18) | 0.396 |  |
|  | Presubiculum | 0.88(0.76;1.01) | 0.070 |  | 0.88(0.76;1.02) | 0.082 |  |
|  | Parasubiculum | 1.03(0.92;1.15) | 0.649 |  | 1.04(0.92;1.17) | 0.536 |  |
|  | Molecular layer | 1.15(0.99;1.33) | 0.067 |  | 1.09(0.94;1.27) | 0.264 |  |
|  | Dentate gyrus | **0.78(0.62;0.97)** | **0.028** |  | 0.82(0.66;1.03) | 0.092 |  |
|  | CA3 | 1.06(0.92;1.22) | 0.398 |  | 1.10(0.95;1.27) | 0.216 |  |
|  | CA4 | 0.99(0.77;1.26) | 0.913 |  | 1.04(0.81;1.33) | 0.771 |  |
|  | Fimbria | **0.85(0.75;0.97)** | **0.015** |  | **0.86(0.75;0.98)** | **0.026** |  |
|  | HATA | 0.88(0.77;1.01) | 0.060 |  | 0.88(0.77;1.01) | 0.074 |  |
| RH | Total HC | 1.05(0.91;1.22) | 0.493 |  | 1.06(0.91;1.22) | 0.467 |  |
|  | Tail | 1.03(0.87;1.21) | 0.754 |  | 1.03(0.88;1.22) | 0.703 |  |
|  | Subiculum | 0.84(0.69;1.01) | 0.063 |  | 0.84(0.69;1.02) | 0.071 |  |
|  | CA1 | 1.18(0.91;1.52) | 0.213 |  | 1.18(0.91;1.53) | 0.202 |  |
|  | Fissure | 1.08(0.96;1.22) | 0.194 |  | 1.05(0.93;1.18) | 0.456 |  |
|  | Presubiculum | 0.94(0.81;1.08) | 0.365 |  | 0.94(0.82;1.09) | 0.407 |  |
|  | Parasubiculum | 0.99(0.88;1.11) | 0.878 |  | 1.00(0.89;1.12) | 0.992 |  |
|  | Molecular layer | **1.22(1.04;1.42)** | **0.015** |  | 1.16(0.99;1.36) | 0.073 |  |
|  | Dentate gyrus | 0.92(0.74;1.13) | 0.415 |  | 0.94(0.76;1.16) | 0.567 |  |
|  | CA3 | 0.92(0.80;1.06) | 0.264 |  | 0.94(0.81;1.09) | 0.403 |  |
|  | CA4 | 0.99(0.80;1.22) | 0.895 |  | 0.99(0.79;1.23) | 0.897 |  |
|  | Fimbria | 1.00(0.88;1.13) | 0.993 |  | 0.99(0.87;1.12) | 0.838 |  |
|  | HATA | 0.97(0.85;1.11) | 0.651 |  | 0.97(0.85;1.11) | 0.689 |  |

*Table shows the results of Cox regression analyses predicting risk of incident clinical depression. Results are presented in hazard ratios (HR) and confidence intervals (CI). Bold shows nominally significant (p<0.05); Star (*) shows multiple comparison correction significant (p<0.0039). Abbreviations: LH/RH, Left/right hemisphere; HC, Hippocampus; CA, Cornu Ammonis; Dentate gyrus, HATA, Hippocampus-amygdala-transition-area.*

***Supplementary Table S8|*** *Results, course of incident depression (chronic or transient)*

|  |  | Main | | | A) One incident event | | | B) One or more incident events | | |
| --- | --- | --- | --- | --- | --- | --- | --- | --- | --- | --- |
|  | **Structure** | **HR(95%CI)** | **P value** |  | **HR(95%CI)** | **P value** |  | **HR(95%CI)** | **P value** |  |
| LH | Total HC | 0.96(0.83;1.12) | 0.644 |  | 0.87(0.72;1.04) | 0.127 |  | 1.22(0.93;1.60) | 0.161 |  |
|  | Tail | 1.15(0.99;1.35) | 0.074 |  | 1.10(0.91;1.33) | 0.324 |  | 1.29(0.98;1.69) | 0.069 |  |
|  | Subiculum | 0.83(0.69;1.01) | 0.057 |  | 0.95(0.75;1.19) | 0.631 |  | **0.62(0.44;0.87)** | **0.005** |  |
|  | CA1 | 1.09(0.85;1.39) | 0.483 |  | 0.99(0.74;1.33) | 0.955 |  | 1.36(0.89;2.10) | 0.159 |  |
|  | Fissure | 1.05(0.94;1.18) | 0.396 |  | 1.00(0.87;1.16) | 0.967 |  | 1.17(0.96;1.43) | 0.122 |  |
|  | Presubiculum | 0.88(0.76;1.02) | 0.082 |  | 0.95(0.80;1.13) | 0.573 |  | **0.75(0.58;0.96)** | **0.024** |  |
|  | Parasubiculum | 1.04(0.92;1.17) | 0.536 |  | 1.01(0.88;1.16) | 0.895 |  | 1.10(0.89;1.35) | 0.366 |  |
|  | Molecular layer | 1.09(0.94;1.27) | 0.264 |  | 1.08(0.90;1.31) | 0.402 |  | 1.12(0.85;1.48) | 0.418 |  |
|  | Dentate gyrus | 0.82(0.66;1.03) | 0.092 |  | 0.80(0.61;1.06) | 0.119 |  | 0.83(0.56;1.24) | 0.366 |  |
|  | CA3 | 1.10(0.95;1.27) | 0.216 |  | 1.04(0.87;1.24) | 0.660 |  | 1.22(0.95;1.57) | 0.127 |  |
|  | CA4 | 1.04(0.81;1.33) | 0.771 |  | 0.95(0.70;1.28) | 0.726 |  | 1.21(0.79;1.87) | 0.381 |  |
|  | Fimbria | **0.86(0.75;0.98)** | **0.026** |  | 0.91(0.77;1.06) | 0.230 |  | **0.76(0.61;0.97)** | **0.024** |  |
|  | HATA | 0.88(0.77;1.01) | 0.074 |  | 0.99(0.84;1.17) | 0.938 |  | **0.70(0.55;0.89)** | **0.003** | ***** |
| RH | Total HC | 1.06(0.91;1.22) | 0.467 |  | 1.00(0.84;1.20) | 0.986 |  | 1.19(0.92;1.54) | 0.187 |  |
|  | Tail | 1.03(0.88;1.22) | 0.703 |  | 1.01(0.83;1.23) | 0.930 |  | 1.09(0.82;1.45) | 0.563 |  |
|  | Subiculum | 0.84(0.69;1.02) | 0.071 |  | 0.89(0.71;1.13) | 0.344 |  | 0.72(0.52;1.01) | 0.057 |  |
|  | CA1 | 1.18(0.91;1.53) | 0.202 |  | 1.04(0.76;1.42) | 0.817 |  | **1.60(1.02;2.52)** | **0.041** |  |
|  | Fissure | 1.05(0.93;1.18) | 0.456 |  | 1.00(0.86;1.16) | 0.989 |  | 1.17(0.95;1.44) | 0.137 |  |
|  | Presubiculum | 0.94(0.82;1.09) | 0.407 |  | 0.98(0.82;1.16) | 0.800 |  | 0.87(0.68;1.11) | 0.249 |  |
|  | Parasubiculum | 1.00(0.89;1.12) | 0.992 |  | 0.95(0.83;1.09) | 0.466 |  | 1.11(0.91;1.34) | 0.306 |  |
|  | Molecular layer | 1.16(0.99;1.36) | 0.073 |  | 1.18(0.98;1.44) | 0.087 |  | 1.12(0.84;1.49) | 0.432 |  |
|  | Dentate gyrus | 0.94(0.76;1.16) | 0.567 |  | 0.96(0.74;1.25) | 0.760 |  | 0.87(0.60;1.28) | 0.488 |  |
|  | CA3 | 0.94(0.81;1.09) | 0.403 |  | 0.95(0.79;1.13) | 0.538 |  | 0.93(0.71;1.21) | 0.592 |  |
|  | CA4 | 0.99(0.79;1.23) | 0.897 |  | 1.03(0.79;1.34) | 0.847 |  | 0.90(0.61;1.32) | 0.581 |  |
|  | Fimbria | 0.99(0.87;1.12) | 0.838 |  | 1.01(0.86;1.17) | 0.937 |  | 0.94(0.75;1.17) | 0.573 |  |
|  | HATA | 0.97(0.85;1.11) | 0.689 |  | 0.96(0.81;1.13) | 0.620 |  | 1.00(0.79;1.26) | 0.986 |  |

*Table shows the results of Cox regression analyses predicting risk of incident clinical depression stratified by number of incident events as* ***A)*** *transient (only one incident event), and* ***B)*** *chronic (more than one incident events) clinically relevant depressive symptoms. Results are presented in hazard ratios (HR) and confidence intervals (CI). Bold shows nominally significant (p<0.05); Star (*) shows multiple comparison correction significant (p<0.0039). Abbreviations: LH/RH, Left/right hemisphere; HC, Hippocampus; CA, Cornu Ammonis; Dentate gyrus, HATA, Hippocampus-amygdala-transition-area.*

***Supplementary Table S9|*** *Sensitivity analysis, prevalent depressive symptoms (PHQ-9 score)*

|  |  | Main | | | A. Exclusion T2DM | | | B. Adjusting for antidepressant medication | | | C. Exclusion antidepressant medication | | | D. Exclusion past depression | | | E. Adjusting for cognition | | | |  |
| --- | --- | --- | --- | --- | --- | --- | --- | --- | --- | --- | --- | --- | --- | --- | --- | --- | --- | --- | --- | --- | --- |
|  | **Structure** | **RR(95%CI)** | **p value** |  | **RR(95%CI)** | **p value** |  | **RR(95%CI)** | **p value** |  | **RR(95%CI)** | **p value** |  | **RR(95%CI)** | **p value** |  | **RR(95%CI)** | **p value** |  | |  |
| LH | Total HC | 0.98(0.94;1.03) | 0.477 |  | 0.99(0.94;1.04) | 0.676 |  | 0.99(0.95;1.04) | 0.751 |  | 1.00(0.95;1.05) | 0.974 |  | 0.98(0.92;1.04) | 0.492 |  | 0.98(0.94;1.03) | 0.519 | |  | |
|  | Tail | 1.02(0.97;1.07) | 0.441 |  | 1.02(0.97;1.08) | 0.463 |  | 1.02(0.97;1.07) | 0.469 |  | 1.01(0.96;1.06) | 0.714 |  | 1.00(0.94;1.06) | 0.916 |  | 1.02(0.97;1.07) | 0.530 | |  | |
|  | Subiculum | 1.03(0.97;1.10) | 0.279 |  | 1.02(0.95;1.09) | 0.577 |  | 1.03(0.97;1.09) | 0.404 |  | 1.03(0.96;1.09) | 0.441 |  | 1.05(0.97;1.13) | 0.216 |  | 1.03(0.97;1.10) | 0.338 | |  | |
|  | CA1 | 1.01(0.93;1.09) | 0.779 |  | 1.03(0.94;1.12) | 0.575 |  | 1.02(0.94;1.10) | 0.638 |  | 1.02(0.94;1.11) | 0.569 |  | 1.06(0.96;1.17) | 0.274 |  | 1.01(0.93;1.10) | 0.798 | |  | |
|  | Fissure | 1.04(1.00;1.08) | 0.054 |  | 1.03(0.98;1.07) | 0.237 |  | 1.03(1.00;1.07) | 0.082 |  | 1.03(0.99;1.07) | 0.143 |  | **1.06(1.01;1.11)** | **0.023** |  | 1.04(1.00;1.08) | 0.083 | |  | |
|  | Presubiculum | 0.98(0.94;1.03) | 0.526 |  | 0.98(0.93;1.03) | 0.426 |  | 0.98(0.94;1.03) | 0.439 |  | 0.99(0.94;1.03) | 0.564 |  | 0.99(0.93;1.05) | 0.691 |  | 0.99(0.94;1.04) | 0.592 | |  | |
|  | Parasubiculum | 0.98(0.94;1.02) | 0.287 |  | 0.97(0.93;1.01) | 0.181 |  | 0.98(0.94;1.02) | 0.288 |  | 0.98(0.94;1.02) | 0.367 |  | 0.99(0.95;1.04) | 0.686 |  | 0.98(0.94;1.02) | 0.330 | |  | |
|  | Molecular layer | **1.06(1.01;1.12)** | **0.029** |  | 1.06(1.00;1.12) | 0.050 |  | 1.05(1.00;1.11) | 0.053 |  | 1.04(0.99;1.10) | 0.112 |  | 1.05(0.98;1.11) | 0.153 |  | 1.06(1.00;1.12) | 0.034 | |  | |
|  | Dentate gyrus | **0.93(0.86;1.00)** | **0.040** |  | 0.92(0.85;1.00) | 0.055 |  | **0.93(0.86;1.00)** | **0.044** |  | 0.94(0.87;1.01) | 0.111 |  | 0.94(0.85;1.03) | 0.147 |  | 0.93(0.86;1.00) | 0.054 | |  | |
|  | CA3 | 0.96(0.91;1.00) | 0.062 |  | 0.97(0.92;1.02) | 0.178 |  | 0.97(0.92;1.01) | 0.136 |  | 0.97(0.92;1.02) | 0.166 |  | 0.95(0.90;1.01) | 0.075 |  | 0.96(0.91;1.00) | 0.065 | |  | |
|  | CA4 | 0.96(0.88;1.04) | 0.261 |  | 0.96(0.87;1.04) | 0.308 |  | 0.96(0.88;1.04) | 0.268 |  | 0.96(0.88;1.05) | 0.354 |  | 0.97(0.88;1.07) | 0.501 |  | 0.95(0.88;1.04) | 0.260 | |  | |
|  | Fimbria | 0.98(0.94;1.03) | 0.436 |  | 0.99(0.94;1.03) | 0.564 |  | 0.99(0.95;1.03) | 0.528 |  | 0.99(0.95;1.03) | 0.656 |  | 0.97(0.92;1.02) | 0.236 |  | 0.99(0.95;1.03) | 0.658 | |  | |
|  | HATA | 1.00(0.96;1.05) | 0.917 |  | 1.01(0.96;1.06) | 0.780 |  | 1.00(0.96;1.05) | 0.948 |  | 1.01(0.96;1.05) | 0.823 |  | 1.01(0.96;1.07) | 0.678 |  | 1.00(0.96;1.05) | 0.901 | |  | |
| RH | Total HC | 0.97(0.92;1.01) | 0.170 |  | 0.97(0.92;1.02) | 0.227 |  | 0.98(0.93;1.03) | 0.391 |  | 0.99(0.94;1.04) | 0.574 |  | 0.97(0.91;1.03) | 0.276 |  | 0.97(0.92;1.02) | 0.232 | |  | |
|  | Tail | 0.99(0.94;1.05) | 0.847 |  | 1.00(0.94;1.06) | 0.980 |  | 0.99(0.94;1.04) | 0.743 |  | 0.98(0.93;1.04) | 0.569 |  | 1.02(0.95;1.09) | 0.574 |  | 0.99(0.94;1.05) | 0.749 | |  | |
|  | Subiculum | 1.05(0.99;1.12) | 0.133 |  | 1.04(0.97;1.11) | 0.233 |  | 1.04(0.98;1.10) | 0.217 |  | 1.04(0.97;1.10) | 0.277 |  | 1.05(0.98;1.14) | 0.185 |  | 1.04(0.98;1.11) | 0.225 | |  | |
|  | CA1 | 1.07(0.98;1.16) | 0.120 |  | 1.06(0.97;1.16) | 0.223 |  | 1.05(0.97;1.14) | 0.226 |  | 1.05(0.96;1.14) | 0.316 |  | 1.04(0.94;1.15) | 0.443 |  | 1.07(0.98;1.16) | 0.143 | |  | |
|  | Fissure | **1.04(1.00;1.08)** | **0.040** |  | 1.05(1.00;1.09) | 0.052 |  | 1.04(1.00;1.08) | 0.054 |  | 1.04(0.99;1.08) | 0.099 |  | **1.05(1.00;1.11)** | **0.037** |  | **1.04(1.00;1.09)** | **0.040** | |  | |
|  | Presubiculum | 1.00(0.96;1.05) | 0.899 |  | 0.99(0.94;1.04) | 0.726 |  | 1.01(0.96;1.05) | 0.828 |  | 1.01(0.96;1.06) | 0.669 |  | 1.03(0.97;1.09) | 0.278 |  | 1.00(0.96;1.05) | 0.970 | |  | |
|  | Parasubiculum | 1.00(0.96;1.04) | 0.976 |  | 1.00(0.96;1.04) | 0.945 |  | 1.00(0.96;1.03) | 0.863 |  | 0.99(0.95;1.03) | 0.744 |  | 1.03(0.98;1.08) | 0.221 |  | 1.00(0.96;1.04) | 0.856 | |  | |
|  | Molecular layer | 1.08(1.02;1.14) | 0.006 |  | **1.08(1.01;1.15)** | **0.016** |  | **1.07(1.01;1.13)** | **0.020** |  | 1.06(1.00;1.12) | 0.064 |  | 1.05(0.98;1.13) | 0.137 |  | **1.08(1.02;1.15)** | **0.005** | |  | |
|  | Dentate gyrus | 0.90(0.84;0.97) | 0.005 |  | **0.92(0.85;0.99)** | **0.026** |  | **0.92(0.86;0.99)** | **0.019** |  | 0.94(0.87;1.01) | 0.090 |  | **0.88(0.81;0.96)** | **0.005** |  | **0.91(0.85;0.98)** | **0.012** | |  | |
|  | CA3 | 0.94(0.89;0.98) | 0.007 |  | 0.95(0.90;1.01) | 0.080 |  | **0.95(0.90;0.99)** | **0.023** |  | 0.95(0.91;1.00) | 0.057 |  | **0.93(0.88;0.99)** | **0.018** |  | **0.94(0.90;0.99)** | **0.016** | |  | |
|  | CA4 | **0.89(0.83;0.96)** | **0.002** | ***** | **0.92(0.85;1.00)** | **0.048** |  | **0.91(0.85;0.98)** | **0.009** |  | **0.93(0.86;1.00)** | **0.045** |  | **0.89(0.81;0.96)** | **0.006** |  | **0.90(0.84;0.97)** | **0.006** | |  | |
|  | Fimbria | 1.00(0.96;1.04) | 0.898 |  | 0.98(0.94;1.03) | 0.476 |  | 1.01(0.97;1.05) | 0.751 |  | 1.01(0.96;1.05) | 0.753 |  | 0.99(0.94;1.04) | 0.633 |  | 1.00(0.96;1.04) | 0.952 | |  | |
|  | HATA | 1.00(0.95;1.04) | 0.939 |  | 0.98(0.93;1.03) | 0.445 |  | 1.00(0.96;1.05) | 0.876 |  | 1.01(0.96;1.06) | 0.763 |  | 0.98(0.93;1.04) | 0.559 |  | 1.00(0.96;1.05) | 0.919 | |  | |

*Sensitivity analyses are performed over Model 2. First column shows main results of Model 2. Panel* ***A)*** *shows the results after the* exclusion of *participants with T2DM (in this case, there is no adjustment for T2DM; panel* ***B)*** *After additionally adjusting Model 2 for antidepressant medication; panel* ***C)*** *after excluding participants using antidepressant medication; and panel* ***D)*** *after excluding participants with a lifetime of major depressive disorder diagnosis. Results are presented in rate ratio (RR) and confidence intervals (CI). Bold shows nominally significant (p<0.05); Star(*) shows multiple comparison correction significant (p<0.0039). Abbreviations: LH/RH, Left/right hemisphere; HC, Hippocampus; CA, Cornu Ammonis; HATA, Hippocampus-amygdala-transition-area.*

***Supplementary Table S10|*** *Sensitivity analysis, prevalent clinically relevant depressive symptoms (PHQ-9≥10)*

|  |  | Main | | | A. Exclusion T2DM | | | B. Adjusting for antidepressant medication | | | C. Exclusion antidepressant medication | | | D. Exclusion past depression | | | E. Adjusting for cognition | | | | |  |
| --- | --- | --- | --- | --- | --- | --- | --- | --- | --- | --- | --- | --- | --- | --- | --- | --- | --- | --- | --- | --- | --- | --- |
|  | **Structure** | **OR(95%CI)** | **p value** |  | **OR(95%CI)** | **p value** |  | **OR(95%CI)** | **p value** |  | **OR(95%CI)** | **p value** |  | **OR(95%CI)** | **p value** |  | **OR(95%CI)** | **p value** | |  | |  |
| LH | Total HC | 0.96(0.78;1.17) | 0.659 |  | 1.01(0.80;1.28) | 0.930 |  | 0.97(0.79;1.19) | 0.787 |  | 1.07(0.85;1.35) | 0.568 |  | 1.11(0.75;1.62) | 0.606 |  | 0.98(0.80;1.20) | | 0.827 | |  | |
|  | Tail | 1.22(0.99;1.50) | 0.063 |  | 1.19(0.93;1.51) | 0.161 |  | 1.23(1.00;1.52) | 0.052 |  | 1.18(0.93;1.49) | 0.183 |  | 0.92(0.62;1.36) | 0.680 |  | 1.23(1.00;1.52) | | 0.050 | |  | |
|  | Subiculum | 1.18(0.92;1.52) | 0.187 |  | 1.13(0.85;1.51) | 0.408 |  | 1.16(0.90;1.50) | 0.244 |  | 1.17(0.88;1.55) | 0.281 |  | 1.53(0.97;2.40) | 0.068 |  | 1.18(0.92;1.52) | | 0.198 | |  | |
|  | CA1 | 0.94(0.67;1.31) | 0.704 |  | 0.98(0.66;1.45) | 0.920 |  | 0.97(0.69;1.36) | 0.857 |  | 0.96(0.66;1.41) | 0.848 |  | 1.06(0.56;2.01) | 0.865 |  | 0.90(0.64;1.27) | | 0.556 | |  | |
|  | Fissure | **1.20(1.03;1.39)** | **0.017** |  | 1.14(0.95;1.37) | 0.169 |  | **1.19(1.02;1.39)** | **0.023** |  | **1.20(1.01;1.42)** | **0.039** |  | **1.30(1.00;1.68)** | **0.049** |  | **1.19(1.02;1.38)** | | **0.026** | |  | |
|  | Presubiculum | 0.92(0.75;1.11) | 0.376 |  | 0.88(0.70;1.11) | 0.295 |  | 0.89(0.73;1.09) | 0.272 |  | 0.95(0.76;1.19) | 0.666 |  | 0.91(0.63;1.32) | 0.609 |  | 0.93(0.76;1.13) | | 0.478 | |  | |
|  | Parasubiculum | **0.82(0.70;0.96)** | **0.014** |  | **0.79(0.65;0.95)** | **0.013** |  | **0.81(0.69;0.95)** | **0.011** |  | **0.78(0.65;0.94)** | **0.008** |  | 0.79(0.58;1.06) | 0.114 |  | **0.82(0.70;0.97)** | | **0.019** | |  | |
|  | Molecular layer | 1.17(0.95;1.44) | 0.146 |  | 1.19(0.94;1.51) | 0.157 |  | 1.16(0.94;1.44) | 0.175 |  | 1.09(0.86;1.38) | 0.488 |  | 1.12(0.76;1.65) | 0.565 |  | 1.15(0.93;1.42) | | 0.195 | |  | |
|  | Dentate gyrus | **0.74(0.54;1.00)** | **0.048** |  | 0.79(0.55;1.12) | 0.179 |  | **0.72(0.53;0.98)** | **0.037** |  | 0.77(0.54;1.09) | 0.142 |  | 0.96(0.53;1.72) | 0.879 |  | **0.73(0.54;0.99)** | | **0.046** | |  | |
|  | CA3 | 0.89(0.73;1.09) | 0.262 |  | 0.92(0.73;1.15) | 0.443 |  | 0.92(0.75;1.12) | 0.383 |  | 0.96(0.77;1.20) | 0.702 |  | 0.86(0.60;1.24) | 0.427 |  | 0.89(0.74;1.09) | | 0.264 | |  | |
|  | CA4 | 0.87(0.63;1.22) | 0.425 |  | 0.93(0.64;1.37) | 0.732 |  | 0.87(0.62;1.21) | 0.398 |  | 0.92(0.63;1.34) | 0.654 |  | 1.11(0.59;2.08) | 0.741 |  | 0.87(0.63;1.21) | | 0.415 | |  | |
|  | Fimbria | 0.91(0.76;1.09) | 0.323 |  | 0.89(0.73;1.10) | 0.292 |  | 0.91(0.76;1.10) | 0.329 |  | 0.92(0.75;1.13) | 0.409 |  | 0.96(0.68;1.34) | 0.800 |  | 0.93(0.78;1.11) | | 0.423 | |  | |
|  | HATA | 0.86(0.72;1.03) | 0.110 |  | 0.93(0.75;1.15) | 0.499 |  | 0.85(0.71;1.03) | 0.094 |  | 0.87(0.70;1.07) | 0.186 |  | 0.99(0.70;1.42) | 0.977 |  | 0.86(0.71;1.03) | | 0.098 | |  | |
| RH | Total HC | 0.90(0.74;1.10) | 0.318 |  | 0.93(0.74;1.18) | 0.568 |  | 0.93(0.76;1.14) | 0.465 |  | 1.02(0.81;1.28) | 0.881 |  | 1.09(0.75;1.57) | 0.661 |  | 0.93(0.76;1.13) | | 0.455 | |  | |
|  | Tail | 1.04(0.83;1.29) | 0.751 |  | 1.01(0.79;1.30) | 0.937 |  | 1.05(0.84;1.31) | 0.684 |  | 0.96(0.75;1.23) | 0.760 |  | 1.19(0.77;1.82) | 0.432 |  | 1.04(0.84;1.30) | | 0.721 | |  | |
|  | Subiculum | **1.38(1.07;1.78)** | **0.014** |  | 1.27(0.95;1.71) | 0.113 |  | **1.34(1.04;1.74)** | **0.026** |  | **1.37(1.03;1.83)** | **0.033** |  | 1.48(0.92;2.38) | 0.108 |  | **1.36(1.05;1.76)** | | **0.018** | |  | |
|  | CA1 | 1.29(0.91;1.83) | 0.154 |  | 1.41(0.93;2.14) | 0.104 |  | 1.24(0.87;1.77) | 0.233 |  | 1.28(0.86;1.91) | 0.228 |  | 1.18(0.62;2.25) | 0.619 |  | 1.24(0.87;1.76) | | 0.240 | |  | |
|  | Fissure | **1.26(1.08;1.48)** | **0.003** | ***** | **1.28(1.06;1.54)** | **0.011** |  | **1.26(1.07;1.48)** | **0.005** |  | **1.25(1.04;1.49)** | **0.015** |  | **1.38(1.04;1.83)** | **0.026** |  | **1.27(1.08;1.48)** | | **0.003** | | ***** | |
|  | Presubiculum | 0.94(0.78;1.14) | 0.518 |  | 0.84(0.67;1.05) | 0.126 |  | 0.93(0.77;1.13) | 0.468 |  | 0.96(0.77;1.19) | 0.710 |  | 0.96(0.67;1.38) | 0.845 |  | 0.95(0.78;1.15) | | 0.572 | |  | |
|  | Parasubiculum | 0.93(0.79;1.09) | 0.357 |  | 0.91(0.75;1.09) | 0.294 |  | 0.93(0.79;1.09) | 0.363 |  | 0.88(0.74;1.05) | 0.166 |  | 1.09(0.81;1.45) | 0.573 |  | 0.92(0.79;1.08) | | 0.322 | |  | |
|  | Molecular layer | **1.31(1.05;1.64)** | **0.015** |  | **1.38(1.06;1.79)** | **0.016** |  | **1.28(1.02;1.60)** | **0.036** |  | 1.17(0.91;1.51) | 0.230 |  | 1.28(0.85;1.92) | 0.238 |  | **1.31(1.05;1.64)** | | **0.017** | |  | |
|  | Dentate gyrus | **0.69(0.51;0.93)** | **0.014** |  | 0.78(0.56;1.11) | 0.167 |  | **0.71(0.52;0.96)** | **0.025** |  | 0.80(0.57;1.13) | 0.206 |  | **0.53(0.30;0.94)** | **0.029** |  | **0.70(0.51;0.94)** | | **0.018** | |  | |
|  | CA3 | **0.75(0.62;0.92)** | **0.006** |  | 0.79(0.63;1.00) | 0.053 |  | **0.78(0.63;0.95)** | **0.015** |  | 0.83(0.66;1.04) | 0.111 |  | 0.73(0.50;1.06) | 0.094 |  | **0.77(0.63;0.94)** | | **0.009** | |  | |
|  | CA4 | **0.68(0.50;0.92)** | **0.013** |  | 0.79(0.56;1.13) | 0.196 |  | **0.70(0.52;0.95)** | **0.024** |  | 0.79(0.56;1.11) | 0.178 |  | **0.56(0.32;0.99)** | **0.048** |  | **0.70(0.52;0.95)** | | **0.022** | |  | |
|  | Fimbria | 0.95(0.80;1.13) | 0.583 |  | 0.92(0.75;1.13) | 0.427 |  | 0.98(0.82;1.17) | 0.817 |  | 0.95(0.78;1.15) | 0.597 |  | 0.90(0.65;1.26) | 0.546 |  | 0.96(0.80;1.14) | | 0.614 | |  | |
|  | HATA | 0.91(0.75;1.09) | 0.287 |  | 0.89(0.72;1.09) | 0.261 |  | 0.92(0.77;1.11) | 0.386 |  | 0.96(0.78;1.19) | 0.725 |  | 0.90(0.63;1.27) | 0.542 |  | 0.90(0.75;1.09) | | 0.279 | |  | |

*Sensitivity analyses are performed over Model 2. First column shows main results of Model 2. Panel* ***A)*** *shows the results after the* exclusion of *participants with T2DM (in this case, there is no adjustment for T2DM; panel* ***B)*** *After additionally adjusting Model 2 for antidepressant medication; panel* ***C)*** *after excluding participants using antidepressant medication; and panel* ***D)*** *after excluding participants with a lifetime of major depressive disorder diagnosis. Results are presented in odds ratio (OR) and confidence intervals (CI). Bold shows nominally significant (p<0.05); Star(*) shows multiple comparison correction significant (p<0.0039). Abbreviations: LH/RH, Left/right hemisphere; HC, Hippocampus; CA, Cornu Ammonis; HATA, Hippocampus-amygdala-transition-area.*

***Supplementary Table S11|*** *Sensitivity analysis, chronic depressive symptoms*

|  |  | Main | | | A. Exclusion T2DM | | | B. Adjusting for antidepressant medication | | | C. Exclusion antidepressant medication | | | D. Exclusion past depression | | E. Adjusting for cognition | | |
| --- | --- | --- | --- | --- | --- | --- | --- | --- | --- | --- | --- | --- | --- | --- | --- | --- | --- | --- |
|  | **Structure** | **OR(95%CI)** | **P value** |  | **OR(95%CI)** | **P value** |  | **OR(95%CI)** | **P value** |  | **OR(95%CI)** | **P value** |  | **OR(95%CI)** | **P value** | **OR(95%CI)** | **P value** |  |
| LH | Total HC | **0.70(0.54;0.91)** | **0.009** |  | 0.75(0.56;1.02) | 0.063 |  | **0.71(0.54;0.93)** | **0.012** |  | 0.76(0.56;1.03) | 0.079 |  | 0.74(0.43;1.26) | 0.266 | **0.73(0.56;0.95)** | **0.019** |  |
|  | Tail | 1.24(0.95;1.63) | 0.114 |  | 1.19(0.87;1.62) | 0.282 |  | 1.27(0.97;1.67) | 0.088 |  | 1.19(0.86;1.65) | 0.282 |  | 1.13(0.64;2.00) | 0.684 | 1.26(0.96;1.65) | 0.096 |  |
|  | Subiculum | 1.17(0.84;1.61) | 0.354 |  | 1.22(0.84;1.77) | 0.293 |  | 1.13(0.81;1.57) | 0.471 |  | 1.08(0.73;1.58) | 0.704 |  | **1.95(1.01;3.76)** | **0.047** | 1.17(0.84;1.62) | 0.351 |  |
|  | CA1 | 1.04(0.68;1.60) | 0.849 |  | 1.03(0.62;1.71) | 0.907 |  | 1.10(0.71;1.71) | 0.671 |  | 1.07(0.64;1.78) | 0.798 |  | 1.14(0.45;2.87) | 0.778 | 1.02(0.66;1.57) | 0.941 |  |
|  | Fissure | **1.37(1.14;1.64)** | **p<0.001** | * | 1.25(1.00;1.58) | 0.055 |  | **1.38(1.14;1.66)** | **p<0.001** | ***** | **1.39(1.13;1.71)** | **0.002** | ***** | **1.49(1.07;2.08)** | **0.018** | **1.35(1.12;1.62)** | **0.002** | ***** |
|  | Presubiculum | 0.86(0.67;1.11) | 0.258 |  | 0.85(0.63;1.14) | 0.287 |  | 0.84(0.65;1.09) | 0.189 |  | 0.92(0.68;1.24) | 0.580 |  | 0.77(0.45;1.32) | 0.350 | 0.88(0.68;1.14) | 0.333 |  |
|  | Parasubiculum | **0.74(0.60;0.91)** | **0.005** |  | **0.74(0.58;0.95)** | **0.017** |  | **0.72(0.58;0.90)** | **0.004** | ***** | **0.68(0.53;0.87)** | **0.003** | ***** | 0.66(0.43;1.01) | 0.058 | **0.75(0.60;0.92)** | **0.007** |  |
|  | Molecular layer | **1.34(1.04;1.74)** | **0.026** |  | **1.36(1.01;1.82)** | **0.042** |  | **1.35(1.03;1.77)** | **0.029** |  | 1.34(1.00;1.80) | 0.053 |  | 1.59(0.99;2.54) | 0.054 | **1.32(1.02;1.72)** | **0.036** |  |
|  | Dentate gyrus | 0.75(0.51;1.12) | 0.159 |  | 0.81(0.51;1.27) | 0.350 |  | 0.73(0.49;1.09) | 0.122 |  | 0.74(0.46;1.18) | 0.200 |  | 0.69(0.29;1.63) | 0.398 | 0.74(0.50;1.10) | 0.135 |  |
|  | CA3 | 0.83(0.65;1.07) | 0.158 |  | 0.81(0.60;1.08) | 0.151 |  | 0.85(0.66;1.10) | 0.220 |  | 0.85(0.63;1.15) | 0.295 |  | 0.65(0.38;1.10) | 0.106 | 0.84(0.65;1.08) | 0.168 |  |
|  | CA4 | 1.02(0.67;1.58) | 0.914 |  | 1.03(0.63;1.70) | 0.894 |  | 1.02(0.66;1.58) | 0.933 |  | 1.02(0.61;1.69) | 0.951 |  | 1.18(0.47;2.96) | 0.728 | 1.00(0.65;1.55) | 0.983 |  |
|  | Fimbria | **0.78(0.61;0.98)** | **0.032** |  | 0.79(0.60;1.03) | 0.082 |  | **0.77(0.61;0.97)** | **0.027** |  | 0.82(0.62;1.08) | 0.151 |  | **0.57(0.34;0.94)** | **0.029** | **0.78(0.62;0.98)** | **0.036** |  |
|  | HATA | 0.81(0.64;1.04) | 0.093 |  | 0.89(0.68;1.17) | 0.410 |  | 0.80(0.63;1.02) | 0.074 |  | 0.90(0.68;1.20) | 0.487 |  | 0.91(0.55;1.53) | 0.731 | 0.80(0.63;1.02) | 0.075 |  |
| RH | Total HC | **0.68(0.52;0.87)** | **0.003** | * | **0.67(0.50;0.91)** | **0.010** |  | **0.69(0.53;0.90)** | **0.007** |  | 0.74(0.55;1.01) | 0.056 |  | 0.80(0.47;1.36) | 0.417 | **0.70(0.54;0.91)** | **0.008** |  |
|  | Tail | 1.14(0.86;1.50) | 0.375 |  | 1.14(0.82;1.57) | 0.431 |  | 1.15(0.87;1.54) | 0.329 |  | 1.05(0.76;1.46) | 0.764 |  | 1.38(0.76;2.51) | 0.290 | 1.15(0.86;1.52) | 0.345 |  |
|  | Subiculum | 1.37(0.99;1.91) | 0.059 |  | 1.32(0.90;1.93) | 0.157 |  | 1.34(0.95;1.88) | 0.096 |  | 1.27(0.86;1.88) | 0.222 |  | **2.08(1.05;4.13)** | **0.036** | 1.36(0.98;1.90) | 0.070 |  |
|  | CA1 | 1.30(0.83;2.03) | 0.252 |  | 1.36(0.80;2.33) | 0.255 |  | 1.25(0.79;1.97) | 0.333 |  | 1.32(0.77;2.24) | 0.309 |  | 1.57(0.63;3.87) | 0.331 | 1.26(0.80;1.98) | 0.326 |  |
|  | Fissure | **1.40(1.15;1.71)** | **p<0.001** | * | **1.37(1.08;1.74)** | **0.009** |  | **1.42(1.16;1.75)** | **p<0.001** | ***** | **1.45(1.15;1.82)** | **0.002** | ***** | **1.73(1.18;2.53)** | **0.005** | **1.42(1.17;1.74)** | **p<0.001** | ***** |
|  | Presubiculum | 1.01(0.79;1.29) | 0.938 |  | 0.89(0.67;1.19) | 0.436 |  | 1.01(0.79;1.29) | 0.939 |  | 1.01(0.76;1.35) | 0.930 |  | 1.02(0.61;1.71) | 0.929 | 1.02(0.80;1.30) | 0.890 |  |
|  | Parasubiculum | 0.87(0.71;1.07) | 0.179 |  | 0.85(0.67;1.07) | 0.168 |  | 0.87(0.71;1.07) | 0.176 |  | 0.80(0.63;1.02) | 0.066 |  | 1.17(0.77;1.77) | 0.459 | 0.86(0.70;1.06) | 0.154 |  |
|  | Molecular layer | **1.51(1.14;2.00)** | **0.004** | * | **1.61(1.16;2.23)** | **0.005** |  | **1.47(1.10;1.97)** | **0.009** |  | **1.40(1.01;1.94)** | **0.046** |  | 1.66(0.95;2.91) | 0.074 | **1.54(1.17;2.04)** | **0.002** | ***** |
|  | Dentate gyrus | **0.63(0.43;0.93)** | **0.019** |  | 0.70(0.45;1.10) | 0.120 |  | **0.64(0.44;0.95)** | **0.027** |  | 0.72(0.46;1.14) | 0.164 |  | **0.33(0.15;0.76)** | **0.009** | **0.62(0.42;0.92)** | **0.017** |  |
|  | CA3 | **0.61(0.48;0.79)** | **p<0.001** | * | **0.65(0.48;0.87)** | **0.004** |  | **0.63(0.49;0.82)** | **p<0.001** | ***** | **0.68(0.51;0.92)** | **0.013** |  | **0.49(0.29;0.82)** | **0.007** | **0.62(0.48;0.80)** | **p<0.001** | ***** |
|  | CA4 | **0.60(0.40;0.88)** | **0.010** |  | 0.68(0.43;1.07) | 0.093 |  | **0.61(0.41;0.91)** | **0.015** |  | 0.68(0.43;1.08) | 0.099 |  | **0.40(0.18;0.90)** | **0.027** | **0.60(0.40;0.89)** | **0.011** |  |
|  | Fimbria | 1.00(0.80;1.24) | 0.973 |  | 0.94(0.73;1.22) | 0.648 |  | 1.03(0.82;1.29) | 0.814 |  | 1.04(0.80;1.35) | 0.775 |  | **0.65(0.41;1.05)** | **0.076** | 1.00(0.80;1.25) | 0.995 |  |
|  | HATA | **0.74(0.59;0.94)** | **0.014** |  | **0.73(0.55;0.96)** | **0.022** |  | **0.77(0.61;0.98)** | **0.036** |  | 0.80(0.60;1.06) | 0.119 |  | 0.70(0.43;1.16) | 0.168 | **0.73(0.58;0.93)** | **0.011** |  |

*Sensitivity analyses are performed over Model 2. First column shows main results of Model 2. Panel* ***A)*** *shows the results after the* exclusion of *participants with T2DM (in this case, there is no adjustment for T2DM; panel* ***B)*** *After additionally adjusting Model 2 for antidepressant medication; panel* ***C)*** *after excluding participants using antidepressant medication; and panel* ***D)*** *after excluding participants with a lifetime of major depressive disorder diagnosis. Results are presented in odds ratio (OR) and confidence intervals (CI). Bold shows nominally significant (p<0.05); Star(*) shows multiple comparison correction significant (p<0.0039). Abbreviations: LH/RH, Left/right hemisphere; HC, Hippocampus; CA, Cornu Ammonis; HATA, Hippocampus-amygdala-transition-area.*

**References**

1. Monereo-Sánchez J, de Jong JJ, Drenthen GS, et al. Quality control strategies for brain MRI segmentation and parcellation: Practical approaches and recommendations-insights from the Maastricht study. *Neuroimage.* 2021;237:118174.
